# The Bayesian Expectation-Maximization-Maximization for the 3PLM

**DOI:** 10.3389/fpsyg.2019.01175

**Published:** 2019-05-31

**Authors:** Shaoyang Guo, Chanjin Zheng

**Affiliations:** ^1^Institute of Curriculum and Instruction, Faculty of Education, East China Normal University, Shanghai, China; ^2^Department of Educational Psychology, Faculty of Education, East China Normal University, Shanghai, China; ^3^Words up your way, Beijing, China

**Keywords:** 3PL, Bayesian EMM, Bayesian EM, mixture modeling, estimation

## Abstract

The current study proposes an alternative feasible Bayesian algorithm for the three-parameter logistic model (3PLM) from a mixture-modeling perspective, namely, the Bayesian Expectation-Maximization-Maximization (Bayesian EMM, or BEMM). As a new maximum likelihood estimation (MLE) alternative to the marginal MLE EM (MMLE/EM) for the 3PLM, the EMM can explore the likelihood function much better, but it might still suffer from the unidentifiability problem indicated by occasional extremely large item parameter estimates. Traditionally, this problem was remedied by the Bayesian approach which led to the Bayes modal estimation (BME) in IRT estimation. The current study attempts to mimic the Bayes modal estimation method and develop the BEMM which, as a combination of the EMM and the Bayesian approach, can bring in the benefits of the two methods. The study also devised a supplemented EM method to estimate the standard errors (SEs). A simulation study and two real data examples indicate that the BEMM can be more robust against the change in the priors than the Bayes modal estimation. The mixture modeling idea and this algorithm can be naturally extended to other IRT with guessing parameters and the four-parameter logistic models (4PLM).

## Introduction

The field of educational testing has witnessed successful development and implementation of a great variety of test item formats, including multiple-choice questions, constructed response questions, and complex performance-based questions. For the past decades, however, multiple-choice questions have been the dominant item format, especially in standardized testing. One major downside of this item format is that examinees may exploit various specific test-taking strategies to improve their performance such as guessing, especially in a low-stakes test (Lord, 1980; Baker and Kim, 2004; Cao and Stokes, 2008; Woods, 2008; Cui et al., 2018).

Consequently, researchers and practitioners have devised powerful statistical tools to model dichotomously scored examinee responses to multiple-choice items. As early as in the nascent stage of IRT, Birnbaum (1968) proposed the three-parameter logistic model (3PLM) and its equivalent model in the normal ogive form to accommodate this need. Since then, the 3PLM has become one of the major statistical tools to analyze multiple-choice data. Various more complicated three-parameter models have been developed and important examples include three-parameter multilevel models (Fox, 2010), three-parameter multidimensional normal ogive model (Samejima, 1974; McDonald, 1999; Bock and Schilling, 2003), three-parameter multidimensional logistic model (Reckase, 2009) and three-parameter partially compensatory multidimensional models (Sympson, 1978).

One major problem that hinders the widespread application of the three-parameter models is the huge challenge of item parameter estimation brought by the guessing parameter. Even for the simplest three-parameter model, the 3PLM, this caused issues for researchers. The marginal maximum likelihood estimation (MMLE) with expectation maximization (EM) algorithm (MMLE/EM) represents the major breakthrough in the estimation techniques for the full-information item factor analysis, but it often runs into convergence problem for data sparseness for the guessing parameter. Mislevy (1986) offered a practical Bayesian solution, namely, the Bayes modal estimation (BME) or the Bayesian EM (BEM), for a moderate sample size, although an MLE solution was not available. Its implementation in BILOG-MG (Zimowski et al., 2003) paved the way for the wide application of the 3PLM in practice. The priors in the Bayesian method can provide extra information and shrink the estimates back to the conditional mean of item parameter, and this shrinkage depends on how informative the priors are. In this case, an informative prior with a smaller variance may have greater influence on the estimation, while a non-informative prior with a larger variance would be relatively weak (Baker and Kim, 2004). However, it is important to emphasize that an informative prior is not the same as an appropriate prior. Mislevy (1986) has warned that, a prior with an incorrect mean and a very small variance is likely to result in a systematic bias. Moreover, when the likelihood function of the MMLE/EM is flat, the Bayesian estimates might be highly dependent on the priors, and lead to a potentially undesirable result. So, it is vital to specify appropriate priors in the Bayesian EM algorithm and one crucial element of BILOG-MG is that the default priors for item parameters are generally uninformative but functional (Mislevy, 1986).

Recently, Zheng et al. (2017) pursued a different direction and developed a more powerful MLE algorithm based on a mixture modeling reformation of 3PLM, namely the Expectation-Maximization-Maximization (EMM). The EMM essentially is modified variant of MMLE/EM and its major difference from the traditional method is to expand the complete data from (**U**, θ) to (**U**, **Z**, θ) where **U** and θ are the response and examinee matrices, and **Z** is the latent indicator for whether some examinees use the guessing strategy for one item. EMM can be summarized conceptually as

a) introducing a new latent variable Z to construct a space one more dimension than the old one, which appears to be unwise because the original 3PLM estimation problem has been made more difficult with one added dimension.

b) invoking the independent assumption of (**Z**, **θ**) to approximate the joint distribution which is one the most commonly used method in statistics to address high-dimension space problems.

c) using the approximation as a surrogate of the original 3PLM likelihood function to obtain item parameter estimate.

Simulation studies indicated that in the expanded space, one can better explore the likelihood function and thus is able to obtain the MLE solution with a moderate sample size. But the EMM is only a partial solution to the possible unidentifiability issue of the 3PLM, evidenced by occasional improbable parameter estimates in the simulation study, and thus further improvement is necessary.

This paper attempts to propose a Bayesian version of the EMM (BEMM) which will bring in the advantages of both the EMM and the Bayesian method. On one hand, the BEMM can solve the unidentifiability issue with the 3PLM by adding additional prior information as in the BEM; on the other hand, it is expected to be more robust against the change in the item parameter priors than the Bayesian EM due to its power in exploitation of the likelihood function.

The rest of the paper is organized as follows: First, the mixture modeling approach to the 3PLM in IRT literature are carefully summarized and an alternative mixture modeling reformulation of the 3PLM with less stringent assumptions is presented. Then, two BEMM algorithms (BEMM-P and BEMM-C) and the estimating method for standard errors (SEs) are derived. One simulation study is carried out to demonstrate the feasibility of the BEMMs, compared to the Bayesian EM and the EMM; and two real data calibrations are presented to show the advantage of the BEMM algorithms. Lastly, future directions are discussed.

## Mixture-Modeling Approach to the 3PLM

Mixture modeling is a powerful statistical tool for accommodating heterogeneity among an overall population. The similar idea for the 3PLM is not even entirely new. A two-process theory on the 3PLM, a p-process which represents the answering behavior based on examinee's ability and a g-process for the guessing strategy, was mentioned by Hutchinson (1991). Later this idea was extensively discussed by San Martín et al. (2006) to justify the development of IRT models of ability-based guessing, and by Maris and Bechger (2009) to demonstrate the identifiability and interpretability issue of the 3PLM. Although von Davier (2009) provided a clear summary of the status quo, differences on the nature of the two processes are far from being settled.

The current paper focus on taking advantage of the two-process reformulation to address the item parameter estimation difficulty of the 3PLM, but the discussion on the conceptual differences is beyond its scope. Two arrangements of these processes can be identified: the g-process comes first or the way around, and thus two different versions of reformulation of the 3PLM can be developed. Interesting enough, the one proposed by Zheng et al. (2017) corresponds to the one with the g-process coming first and Béguin and Glas (2001) proposed an ability-based reformulation for the three-parameter normal ogive model (3PNO) which coincides with the one with the p-process coming first. The two reformulations are briefly reviewed here as the starting point of the two BEMM algorithms

The basic formulation of the 3PLM is defined as:

(1) $$\begin{array}{l} P\left(u_{ij}=1|\theta_{j},a_{i},b_{i},c_{i}\right)\equiv P_{i}\left(\theta_{j}\right)=c_{i}+\frac{1-c_{i}}{1+\text{ exp}\left(-Da_{i}\left(\theta_{j}-b_{i}\right)\right)} \end{array}$$

where *u*_*ij*_ represents the response of examinee *j*(*j* = 1, 2, …, *N*) on item *i*(*i* = 1, 2, …, *n*); *a*_*i*_, *b*_*i*_, and *c*_*i*_ are the discrimination, difficulty, and guessing parameters for the *i*^*th*^ item, respectively; θ_*j*_ is the ability parameter of the examinee*j*; and *D* is the scaling constant, 1.702. Let ξ_*i*_ = (*a*_*i*_, *b*_*i*_, *c*_*i*_) represents the item parameter vector for *i*^*th*^ item, the function *P*(*u*_*ij*_ = 1|θ_*j*_, ξ_*i*_) can be abbreviated as *P*_*i*_(θ_*j*_), which is the probability of the correct response *u*_*ij*_ = 1 to item *i* given θ_*j*_. The 3PLM can be conceived as an extension of the 2PLM with an item-specific guessing parameter:

(2) $$\begin{array}{l} P_{i}\left(\theta_{j}\right)=c_{i}+\left(1-c_{i}\right)\times P_{i}^{*}\left(\theta_{j}\right) \end{array}$$

with

(3) $$\begin{array}{l} P_{i}^{*}\left(\theta_{j}\right)=\frac{1}{1+\exp\left(-Da_{i}\left(\theta_{j}-b_{i}\right)\right)} \end{array}$$

as the 2PLM. Following Béguin and Glas (2001)'s ability-based representation for the three-parameter normal ogive model (3PNO), the 3PLM can also be written as

(4) $$\begin{array}{l} P_{i}\left(\theta_{j}\right)=P_{i}^{*}\left(\theta_{j}\right)+\left[1-P_{i}^{*}\left(\theta_{j}\right)\right]\times c_{i}. \end{array}$$

A reformulation of the ability-based 3PLM, similar to Zheng et al. (2017), can be derived readily. Following Culpepper (2015), we may introduce a latent variable *v*_*ij*_ ∈ **V**, and $v_{ij}\sim \;Bernoulli\left(P_{i}^{*}\left(\theta_{j}\right)\right)$:

$$\begin{array}{l} v_{ij}=\{\begin{array}{c} 1,\text{if examinee }j\text{ has ability to answer item }i\text{ correctly;}\\ 0,\text{if examinee }j\text{does not have ability to answer item }i\text{ correctly.} \end{array} \end{array}$$

Reasonably, *v*_*ij*_ follows a Bernoulli distribution with parameter $P_{i}^{*}\left(\theta_{j}\right)$, or $P\left(v_{ij}=1,{\text{u}}_{ij}=\text{1}|\;\xi_{i},\theta_{j}\right)=P\left({\text{u}}_{ij}=\text{1}|\;\xi_{i},\theta_{j}\right)=P_{i}^{*}\left(\theta_{j}\right)$. From the mixture-modeling perspective, depending on the value of *v*_*ij*_, the possibilities of 3PLM can be decomposed into two irrelevant parts: 1 and *c*_*i*_.

Furthermore, the conditional possibilities *P*(*u*_*ij*_|*v*_*ij*_, θ_*j*_, ***ξ***_*i*_) can be easily obtained as:

(5) $$\begin{array}{l} \begin{array}{c} P\left(u_{ij}=1|v_{ij}=1,\theta_{j},\xi_{i}\right)=1,\;P\left(u_{ij}=1|v_{ij}=0,\theta_{j},\xi_{i}\right)=c_{i},\\ P\left(u_{ij}=0|v_{ij}=1,\theta_{j},\xi_{i}\right)=0,\;P\left(u_{ij}=0|v_{ij}=0,\theta_{j},\xi_{i}\right)=1-c_{i}. \end{array} \end{array}$$

By the multiplication rule, *P*(*u*_*ij*_, *v*_*ij*_|θ_*j*_, ξ_*i*_) = *P*(*u*_*ij*_|*v*_*ij*_, θ_*j*_, ξ_*i*_)*P*(*v*_*ij*_), the joint distribution of *u*_*ij*_ and *v*_*ij*_ can be calculated as:

(6) $$\begin{array}{l} \begin{array}{c} P\left(u_{ij}=1,v_{ij}=1|\theta_{j},\xi_{i}\right)=P_{i}^{*}\left(\theta_{j}\right),\;\\ P\left(u_{ij}=1,v_{ij}=0|\theta_{j},\xi_{i}\right)=c_{i}\left(1-P_{i}^{*}\left(\theta_{j}\right)\right),\\ P\left(u_{ij}=0,v_{ij}=1|\theta_{j},\xi_{i}\right)=0,\;\\ P\left(u_{ij}=0,v_{ij}=0|\theta_{j},\xi_{i}\right)=\left(1-c_{i}\right)\left(1-P_{i}^{*}\left(\theta_{j}\right)\right). \end{array} \end{array}$$

Note that $P_{i}^{*}\left(\theta_{j}\right)+c_{i}\left(1-P_{i}^{*}\left(\theta_{j}\right)\right)+\left(1-c_{i}\right)\left(1-P_{i}^{*}\left(\theta_{j}\right)\right)=1$, so *P*(*u*_*ij*_ = 0, *v*_*ij*_ = 1|θ_*j*_, ξ_*i*_) = 0 is actually redundant and can be omitted from the probability density function. From this distribution law of (*u*_*ij*_, *v*_*ij*_) conditional on(θ_*j*_, **ξ**_*i*_), the marginal likelihood function of the EMM is

(7) $$\begin{array}{c} L\left(U,V|\xi \right)=\prod_{j=1}^{N}\int_{\theta_{j}}\{\prod_{i=1}^{n}P_{i}^{*}{\left(\theta_{j}\right)}^{u_{ij}v_{ij}}\times {\left[c_{i}\left(1-P_{i}^{*}\left(\theta_{j}\right)\right)\right]}^{u_{ij}\left(1-v_{ij}\right)}\\ \;\times {\left[\left(1-c_{i}\right)\left(1-P_{i}^{*}\left(\theta_{j}\right)\right)\right]}^{\left(1-u_{ij}\right)\left(1-v_{ij}\right)}\}g\left(\theta_{j}|\tau \right)d\theta_{j}. \end{array}$$

where **U** is the *n* × *N* response matrix with *u*_*ij*_ as its elements; **V** is defined, with respect to *v*_*ij*_, in analogy to **U** and *u*_*ij*_; ξ = (ξ_1_, ξ_2_, …, ξ_*i*_, …, ξ_*n*_) is the matrix for item parameters; *g*(θ_*j*_|τ) is a density function for examinees' ability, and **τ** is the vector containing the parameters of the examinee population ability distribution.

Zheng et al. (2017) proposed the EMM algorithm based on the new likelihood function derived from the reformulation with the g-process coming first (see Appendix A). The EMM can solve the convergence problem of MMLE/EM in a modest sample size of about 1,000 examinees, but it cannot eliminate occasional improbably large estimates. In fact, this is still a symptom of the 3PLM being possibly unidentifiable, though much more alleviated than in the original MMLE/EM, and this can be resolved by additional prior information which leads to the Bayesian EMM algorithms.

Please note that Béguin and Glas (2001) did not develop an EM algorithm similar to the EMM for the 3PNO because the integral in the ogive model introduces additional difficulty for the E-step which can be conveniently addressed by a MCMC algorithm. An EM algorithm and its variants are a much more natural alternative for the 3PLM which is the main topic of the current paper.

## The Bayesian EMM Algorithms

Take the first reformulation as an example to illustrate how to derive the BEMM algorithm. Following Mislevy (1986)'s parameterization to take logarithmic form of *a*_*i*_, the 3PLM can be rewritten as:

(8) $$\begin{array}{l} p_{i}\left(\theta_{j}\right)=c_{i}+\left(1-c_{i}\right)\times p_{i}^{*}\left(\theta_{j}\right), \end{array}$$

with

(9) $$\begin{array}{l} p_{i}^{*}\left(\theta_{j}\right)=\frac{1}{1+\exp\left(-De^{\ln\;a_{i}}\left(\theta_{j}-b_{i}\right)\right)}. \end{array}$$

Mislevy (1986) also has given a general Bayesian formulation for the 3PLM which we may apply to the BEMM as well. Let ψ_*i*_ represents any item parameter for item *i* in ***ξ***_*i*_, and then the first derivative of the general Bayesian formulation for each item parameter can be obtained as:

(10) $$\begin{array}{l} \frac{\partial \ln\;L}{\partial \psi_{i}}+\frac{\partial \ln\;g\left(\psi_{i}|\eta \right)}{\partial \psi_{i}}, \end{array}$$

with

(11) $$\begin{array}{l} L\left(U,V|\xi \right)=\prod_{j=1}^{N}\int_{\theta_{j}}\{\prod_{i=1}^{n}P_{i}^{*}{\left(\theta_{j}\right)}^{u_{ij}v_{ij}}\times {\left[c_{i}\left(1-P_{i}^{*}\left(\theta_{j}\right)\right)\right]}^{u_{ij}\left(1-v_{ij}\right)}\\ \;\times {\left[\left(1-c_{i}\right)\left(1-P_{i}^{*}\left(\theta_{j}\right)\right)\right]}^{\left(1-u_{ij}\right)\left(1-v_{ij}\right)}\}g\left(\theta_{j}|\tau \right)d\theta_{j} \end{array}$$

where *L* is the likelihood of the EMM (Equation 7) with the logarithmic form of *a*_*i*_, and *g*(ψ_*i*_|η) is the item parameter prior distribution for item *i*. Mislevy (1986) suggested that ln *a*_*i*_ and *b*_*i*_ follow a normal distribution and *c*_*i*_ a beta distribution, specifically,

(12) $$\begin{array}{l} \ln\;a_{i}\sim \;N\left(\mu_{\text{ln }a_{i}},\sigma_{\text{ln }a_{i}}^{2}\right),\;b_{i}\sim \;N\left(\mu_{b_{i}},\sigma_{b_{i}}^{2}\right),\;c_{i}\sim \;Beta\left(\alpha_{i},\beta_{i}\right) \end{array}$$

in which $\mu_{\text{ln }a_{i}},\mu_{b_{i}},\sigma_{\text{ln }a_{i}}^{2},\text{and }\sigma_{b_{i}}^{2}$ are the means and variances for the corresponding normal distribution and α_*i*_, β_*i*_ are the parameters for the beta distribution for the guessing parameter. They may be specified as in the BILOG-MG default setting (Du Toit, 2003):

(13) $$\begin{array}{l} \ln\;a\sim \;N\left(0,0.5^{2}\right),b\sim \;N\left(0,2^{2}\right),c\sim \;Beta\left(4,16\right). \end{array}$$

The first and second derivatives for the three priors are given by Mislevy (1986) as:

(14) $$\begin{array}{l} \begin{array}{c} \frac{\partial \ln\;g\left(\text{ln }a_{i}|\mu_{\text{ln }a_{i}},\sigma_{\text{ln }a_{i}}^{2}\right)}{\partial \text{ln }a_{i}}=-\frac{\ln\;a_{i}-\mu_{\text{ln }a_{i}}}{\sigma_{\text{ln }a_{i}}^{2}},\;\frac{\partial^{2}\ln\;g\left(\text{ln }a_{i}|\mu_{\text{ln }a_{i}},\sigma_{\text{ln }a_{i}}^{2}\right)}{\partial \text{ln }a_{i}\partial \text{ln }a_{i}}=-\frac{1}{\sigma_{\text{ln }a_{i}}^{2}},\\ \frac{\partial \ln\;g\left(b_{i}|\mu_{b_{i}},\sigma_{b_{i}}^{2}\right)}{\partial b_{i}}=-\frac{b_{i}-\mu_{b_{i}}}{\sigma_{b_{i}}^{2}},\;\frac{\partial^{2}\ln\;g\left(b_{i}|\mu_{b_{i}},\sigma_{b_{i}}^{2}\right)}{\partial b_{i}\partial b_{i}}=-\frac{1}{\sigma_{b_{i}}^{2}},\\ \frac{\partial \ln\;g\left(c_{i}|\alpha_{i},\beta_{i}\right)}{\partial c_{i}}=\frac{\alpha_{i}-1}{c_{i}}-\frac{\beta_{i}-1}{1-c_{i}},\;\frac{\partial^{2}\ln\;g\left(c_{i}|\alpha_{i},\beta_{i}\right)}{\partial c_{i}\partial c_{i}}=-\frac{\alpha_{i}-1}{c_{i}^{2}}-\frac{\beta_{i}-1}{{\left(1-c_{i}\right)}^{2}}. \end{array} \end{array}$$

With the prior distribution component explained, the next will describe the Bayesian EMM method in which the likelihood component will be carefully delineated.

### Expectation Step and Artificial Data

The expectation step boils down to the calculation of the conditional expectations of **V** and θ. From the joint distribution in Equation (5), one can calculate the expectation of *v*_*ij*_ conditional on *u*_*ij*_ and the marginal distribution of *v*_*ij*_. By the Bayesian rule,

(15) $$\begin{array}{l} \begin{array}{c} P\left(v_{ij}=1|u_{ij}=1,\theta_{j},\xi_{i}\right)=\frac{P_{i}^{*}\left(\theta_{j}\right)}{P_{i}\left(\theta_{j}\right)},\\ P\left(v_{ij}=1|u_{ij}=0,\theta_{j},\xi_{i}\right)=0 \end{array} \end{array}$$

can be yielded from Equations (5, 6). Then, the conditional expectation of *v*_*ij*_ is

(16) $$\begin{array}{l} E\left(v_{ij}|u_{ij},\theta_{j},\xi_{i}\right)=\frac{P_{i}^{*}\left(\theta_{j}\right)}{P_{i}\left(\theta_{j}\right)}\times u_{ij}+0\times \left(1-u_{ij}\right). \end{array}$$

As for **θ**, by using summation over a fixed grid of equally-spaced quadrature points *X*_*k*_ (*k* = 1, 2, …, *q*) with an associated weight *A*(*X*_*k*_) to approximate integration, one can have the quadrature form of the first derivative of the expected log-likelihood function for each item parameter (see Appendix B for detail):

(17) $$\begin{array}{l} \frac{\partial \ln\;E\left[L\right]}{\partial \psi_{i}}\approx \overset{N}{\underset{j=1}{\sum }}\overset{q}{\underset{k=1}{\sum }}\left[\begin{array}{c} \frac{u_{ij}E\left(v_{ij}|u_{ij},X_{k},\xi_{i}\right)}{P_{i}^{*}\left(X_{k}\right)}\frac{\partial P_{i}^{*}\left(X_{k}\right)}{\partial \psi_{i}}\\ -\frac{1-E\left(v_{ij}|u_{ij},X_{k},\xi_{i}\right)}{1-P_{i}^{*}\left(X_{k}\right)}\frac{\partial P_{i}^{*}\left(X_{k}\right)}{\partial \psi_{i}}\\ +\frac{u_{ij}\left(1-E\left(v_{ij}|u_{ij},X_{k},\xi_{i}\right)\right)}{c_{i}}\frac{\partial c_{i}}{\partial \psi_{i}}\\ -\frac{\left(1-u_{ij}\right)\left(1-E\left(v_{ij}|u_{ij},X_{k},\xi_{i}\right)\right)}{1-c_{i}}\frac{\partial c_{i}}{\partial \psi_{i}} \end{array}\right]P\left(X_{k}\;|\;{\text{u}}_{j},{\text{v}}_{j},\tau ,\xi \right) \end{array}$$

with

(18) $$\begin{array}{l} \begin{array}{c} P\left(X_{k}\;|\;{\text{u}}_{j},{\text{v}}_{j},\tau ,\xi \right)=P\left(X_{k}\;|\;{\text{u}}_{j},\tau ,\xi \right)\text{=}\frac{P\left({\text{u}}_{j}|X_{k},\xi \right)\times A\left(X_{k}\right)}{\sum_{k=1}^{q}P\left({\text{u}}_{j}|X_{k},\xi \right)\times A\left(X_{k}\right)},\\ P\left({\text{u}}_{j}|X_{k},\xi \right)=\prod_{i=1}^{n}P_{i}{\left(X_{k}\right)}^{u_{ij}}\times {\left(1-P_{i}\left(X_{k}\right)\right)}^{1-u_{ij}}, \end{array} \end{array}$$

where *P*(*X*_*k*_|**u**_*j*_, **v**_*j*_, **τ**, **ξ**) is the posterior probability of θ_*j*_ evaluated at *X*_*k*_ given **u**_*j*_, **v**_*j*_, **τ**, and **ξ**. Then, *P*(*X*_*k*_|**u**_*j*_, **v**_*j*_, **τ**, **ξ**) equals *P*(*X*_*k*_|**u**_*j*_, **τ**, **ξ**) because $P\left({\text{u}}_{ij}=\text{1}|\;\xi_{i},\theta_{j}\right)=P\left(v_{ij}=1,{\text{u}}_{ij}=\text{1}|\;\xi_{i},\theta_{j}\right)=P_{i}^{*}\left(\theta_{j}\right)$. Furthermore, *P*(*X*_*k*_|**u**_*j*_, **v**_*j*_, **τ**, **ξ**) can be used to compute the “artificial data”. The “artificial data” is essentially various expected frequencies of examinees under the posterior probability of θ_*j*_ and can be expressed as different linear combinations of the posterior probability of θ_*j*_. Since it is “created” from the posterior probability, the IRT literature terms them as the “artificial data”. For instance, Bock and Aitkin (1981) has provided two fundamental artificial data for traditional EM algorithm as:

(19) $$\begin{array}{l} \begin{array}{c} {\bar{f}}_{k}=\sum_{j=1}^{N}P\left(X_{k}\;|\;{\text{u}}_{j},\tau ,\xi \right)=\sum_{j=1}^{N}P\left(X_{k}\;|\;{\text{u}}_{j},{\text{z}}_{j},\tau ,\xi \right),\\ {\bar{r}}_{ik}=\sum_{j=1}^{N}u_{ij}\times P\left(X_{k}\;|\;{\text{u}}_{j},\tau ,\xi \right)=\sum_{j=1}^{N}u_{ij}\times P\left(X_{k}\;|\;{\text{u}}_{j},{\text{z}}_{j},\tau ,\xi \right), \end{array} \end{array}$$

in which ${\bar{f}}_{k}$ is the expected number of examinees with ability *X*_*k*_. Thus, the sum of ${\bar{f}}_{k}$ for every ability point *X*_*k*_ equals the total number of examinees *N*, and ${\bar{r}}_{ik}$ is the expected number of examinees with ability *X*_*k*_ answering item *i* correctly.

Then, as can be seen from Table 1, the EMM algorithm introduced a new latent variable **V**, so there are two new artificial data as

(20) $$\begin{array}{l} \begin{array}{c} {\bar{f}}_{ik}^{\left(\text{V}\right)}={\bar{r}}_{ik}^{\left(\text{V}\right)}\\ \;=\sum_{j=1}^{N}E\left(v_{ij}|u_{ij},X_{k},\xi \right)P\left(X_{k}\;|\;{\text{u}}_{j},{\text{v}}_{j},\tau ,\xi \right), \end{array} \end{array}$$

in which ${\bar{f}}_{ik}^{\left(\text{V}\right)}$ is the expected number of examinees with ability *X*_*k*_ who have employed their ability to answer item *i* and ${\bar{r}}_{ik}^{\left(\text{V}\right)}$ is the expected number of examinees with ability *X*_*k*_ who are able to answer it correctly. Please note that the expected number of examinees with ability *X*_*k*_ who have answered the item *i* based on their ability but incorrectly is zero, so ${\bar{f}}_{ik}^{\left(\text{V}\right)}={\bar{r}}_{ik}^{\left(\text{V}\right)}$. Moreover, it is easy to obtain the expected number of examinees with ability *X*_*k*_ who have not employed their ability (in another words, used the guessing strategy) to answer item *i*, ${\bar{f}}_{k}-{\bar{f}}_{ik}^{\left(\text{V}\right)}$, and that of examinees among them who are able to answer it correctly, ${\bar{r}}_{ik}-{\bar{r}}_{ik}^{\left(\text{V}\right)}$.

**Table 1:** The definition of four kinds of artificial data.

**Item *i***	****v_*i*_ = 1****	****v_*i*_ = 0****	**Marginal of **v**_***i***_**
**u**_*i*_=1	${\bar{r}}_{ik}^{\left(\text{V}\right)}$	${\bar{r}}_{ik}^{\;}-{\bar{r}}_{ik}^{\left(\text{V}\right)}$	${\bar{r}}_{ik}$
**u**_*i*_=0	0	${\bar{f}}_{k}-{\bar{r}}_{ik}^{\;}$	
Marginal of **u**_*i*_	${\bar{f}}_{k}^{\left(\text{V}\right)}$	${\bar{f}}_{k}-{\bar{f}}_{ik}^{\left(\text{V}\right)}$	${\bar{f}}_{k}$

*Artificial data: The expected number of examinees*.

After the E-step and calculation of the artificial data, the next steps are to compute the first and second derivatives of Equation (17) with respect to each item parameter.

### Maximization Step-1 for *c* Parameter

From Equation (17), the first derivative for the guessing parameter is

(21) $$\begin{array}{l} \begin{array}{c} \lambda_{c_{i}}^{\;}=\frac{\partial \ln\;E\left[L\right]}{\partial c_{i}}+\frac{\partial \ln\;g\left(c_{i}|\alpha_{i},\beta_{i}\right)}{\partial c_{i}}\\ \;\approx \frac{\alpha_{i}-1}{c_{i}}-\frac{\beta_{i}-1}{1-c_{i}}+\sum_{j=1}^{N}\sum_{k=1}^{q}\\ \;\left[\frac{u_{ij}\left(1-E\left(v_{ij}|u_{ij},X_{k},\xi_{i}\right)\right)}{c_{i}}-\frac{\left(1-u_{ij}\right)\left(1-E\left(v_{ij}|u_{ij},X_{k},\xi_{i}\right)\right)}{1-c_{i}}\right]P\left(X_{k}\;|\;{\text{u}}_{j},{\text{z}}_{j},\tau ,\xi \right)\\ \;=\frac{\alpha_{i}-1}{c_{i}}-\frac{\beta_{i}-1}{1-c_{i}}+\frac{\sum_{k=1}^{q}\left({\bar{r}}_{ik}^{\;}-{\bar{r}}_{ik}^{\left(\text{V}\right)}\right)}{c_{i}}-\frac{\sum_{k=1}^{q}\left({\bar{f}}_{k}^{\;}-{\bar{r}}_{ik}^{\;}\right)}{1-c_{i}} \end{array} \end{array}$$

Set Equation (21) to 0 and solve for the estimate of *c*_*i*_ which leads to a closed solution. The derivation is as follows:

(22) $$\begin{array}{l} \begin{array}{c} \Rightarrow \frac{\alpha_{i}-1+\sum_{k=1}^{q}\left({\bar{r}}_{ik}^{\;}-{\bar{r}}_{ik}^{\left(\text{V}\right)}\right)}{c_{i}}=\frac{\beta_{i}-1+\sum_{k=1}^{q}\left({\bar{f}}_{k}^{\;}-{\bar{r}}_{ik}^{\;}\right)}{1-c_{i}}\\ \Rightarrow c_{i}=\frac{\alpha_{i}-1+\sum_{k=1}^{q}\left({\bar{r}}_{ik}^{\;}-{\bar{r}}_{ik}^{\left(\text{V}\right)}\right)}{\alpha_{i}+\beta_{i}-2+\sum_{k=1}^{q}\left({\bar{r}}_{ik}^{\;}-{\bar{r}}_{ik}^{\left(\text{V}\right)}+{\bar{f}}_{k}^{\;}-{\bar{r}}_{ik}^{\;}\right)}\\ \;=\frac{\alpha_{i}-1+\sum_{k=1}^{q}\left({\bar{r}}_{ik}^{\;}-{\bar{r}}_{ik}^{\left(\text{V}\right)}\right)}{\alpha_{i}+\beta_{i}-2+\sum_{k=1}^{q}\left({\bar{f}}_{k}^{\;}-{\bar{f}}_{ik}^{\left(\text{V}\right)}\right)} \end{array} \end{array}$$

The estimate for the guessing parameter is contributed by two components: the prior and the data. The magnitude of the prior parameters α_*i*_ and β_*i*_ determines the influence of the prior through the two terms (α_*i*_ − 1) and (β_*i*_ − 1). By ignoring the prior terms (α_*i*_ − 1) and (β_*i*_ − 1), the data component of the estimate offers a very intuitive interpretation of the guessing parameter: It is calculated as the proportion of examinees who answer item *i* correctly using the guessing strategy in the total sample. This interpretation nicely fits into general philosophy of mixture modeling, drastically different from the traditional interpretation which is defined as the lower bound for the probability with which an examinee answers an item correctly.

Since an analytical solution can be easily obtained, the calculation of the corresponding second derivative and implementation of Newton-Raphson or Fisher-scoring algorithm, as in the traditional EM algorithm, are unnecessary. However, the second derivative is still useful for estimating SEs and is given below:

(23) $$\begin{array}{l} \begin{array}{c} \lambda_{cc_{i}}^{\;}=E\left[\frac{\partial^{2}\ln\;E\left[L\right]}{\partial^{2}c_{i}^{2}}|u_{ij}\right]+\frac{\partial^{2}\ln\;g\left(c_{i}|\alpha_{i},\beta_{i}\right)}{\partial^{2}c_{i}^{2}}\\ \;\approx E\left[\frac{\partial }{\partial c_{i}}\overset{q}{\underset{k=1}{\sum }}\left[\frac{\left({\bar{r}}_{ik}^{\;}-{\bar{r}}_{ik}^{\left(\text{V}\right)}\right)}{c_{i}}-\frac{\left({\bar{f}}_{k}^{\;}-{\bar{r}}_{ik}^{\;}\right)}{1-c_{i}}\right]|u_{ij}\right]-\frac{\alpha_{i}-1}{c_{i}^{2}}-\frac{\beta_{i}-1}{{\left(1-c_{i}\right)}^{2}}\\ \;=E\left[-\frac{\sum_{k=1}^{q}\left({\bar{r}}_{ik}^{\;}-{\bar{r}}_{ik}^{\left(\text{V}\right)}\right)}{c_{i}^{2}}-\frac{\sum_{k=1}^{q}\left({\bar{f}}_{k}^{\;}-{\bar{r}}_{ik}^{\;}\right)}{{\left(1-c_{i}\right)}^{2}}|u_{ij}\right]-\frac{\alpha_{i}-1}{c_{i}^{2}}-\frac{\beta_{i}-1}{{\left(1-c_{i}\right)}^{2}}\\ \;=-\frac{\sum_{k=1}^{q}\left(1-p_{i}^{*}\left(X_{k}\right)\right){\bar{f}}_{k}^{\;}}{c_{i}^{\;}\left(1-c_{i}\right)}-\frac{\alpha_{i}-1}{c_{i}^{2}}-\frac{\beta_{i}-1}{{\left(1-c_{i}\right)}^{2}} \end{array} \end{array}$$

since

(24) $$\begin{array}{l} E\left[u_{ij}|X_{k}\right]=p_{i}\left(X_{k}\right)\\ E\left[E\left(v_{ij}|u_{ij},X_{k},\xi \right)|u_{ij}\right]=E\left(v_{ij}\right)=p_{i}^{*}\left(X_{k}\right) \end{array}$$

and

(25) $$\begin{array}{l} \begin{array}{c} E\left[{\bar{f}}_{k}^{\;}|u_{ij}\right]=E\left[\sum_{j=1}^{N}P\left(X_{k}\;|\;{\text{u}}_{j},{\text{v}}_{j},\tau ,\xi \right)|u_{ij}\right]=\sum_{j=1}^{N}P\left(X_{k}\;|\;{\text{u}}_{j},{\text{v}}_{j},\tau ,\xi \right)\\ \;={\bar{f}}_{k}^{\;},\\ E\left[{\bar{r}}_{ik}^{\;}|u_{ij}\right]=E\left[\sum_{j=1}^{N}u_{ij}P\left(X_{k}\;|\;{\text{u}}_{j},{\text{v}}_{j},\tau ,\xi \right)|u_{ij}\right]\\ \;=p_{i}^{\;}\left(X_{k}\right)\sum_{j=1}^{N}P\left(X_{k}\;|\;{\text{u}}_{j},{\text{v}}_{j},\tau ,\xi \right)\;\\ \;=p_{i}^{\;}\left(X_{k}\right){\bar{f}}_{k}^{\;},\\ E\left[{\bar{f}}_{ik}^{\left(\text{V}\right)}|u_{ij}\right]=E\left[{\bar{r}}_{ik}^{\left(\text{V}\right)}|u_{ij}\right]=E\left[\sum_{j=1}^{N}E\left(v_{ij}|u_{ij},X_{k},\xi \right)P\left(X_{k}\;|\;{\text{u}}_{j},{\text{v}}_{j},\tau ,\xi \right)|u_{ij}\right]\\ \;=p_{i}^{*}\left(X_{k}\right)\sum_{j=1}^{N}P\left(X_{k}\;|\;{\text{u}}_{j},{\text{v}}_{j},\tau ,\xi \right)\;\\ \;=p_{i}^{*}\left(X_{k}\right){\bar{f}}_{k}^{\;}. \end{array} \end{array}$$

### Maximization Step-2 for *a* and *b* Parameters

The second Maximization step is to execute the Fisher-scoring procedure to obtain estimates forln *a*_*i*_ and *b*_*i*_. The required first derivatives for ln *a*_*i*_ and *b*_*i*_ are

(26) $$\begin{array}{l} \begin{array}{c} \lambda_{a_{i}}^{\;}=De_{\;}^{\ln\;a_{i}}\sum_{k=1}^{q}\left[\left({\bar{r}}_{ik}^{\left(\text{V}\right)}-{\bar{f}}_{k}^{\;}\times p_{i}^{*}\left(X_{k}\right)\right)\left(X_{k}-b_{i}\right)\right]-\frac{\ln\;a_{i}-\mu_{\ln\;a_{i}}}{\sigma_{\ln\;a_{i}}^{2}},\\ \lambda_{b_{i}}^{\;}=-De^{\ln\;a_{i}}\sum_{k=1}^{q}\left[{\bar{r}}_{ik}^{\left(\text{V}\right)}-{\bar{f}}_{k}^{\;}\times p_{i}^{*}\left(X_{k}\right)\right]-\frac{b_{i}-\mu_{i}}{\sigma_{b_{i}}^{2}}. \end{array} \end{array}$$

The corresponding expectation of second derivatives are:

(27) $$\begin{array}{l} \begin{array}{c} \lambda_{aa_{i}}^{\;}=-D^{2}e_{\;}^{2\ln\;a_{i}}\sum_{k=1}^{q}{\left(X_{k}-b_{i}\right)}^{2}w_{ik}^{*}\times {\bar{f}}_{k}^{\;}-\frac{1}{\sigma_{\ln\;a_{i}}^{2}},\\ \lambda_{bb_{i}}^{\;}=-D^{2}e_{\;}^{2\ln\;a_{i}}\sum_{k=1}^{q}w_{ik}^{*}\times {\bar{f}}_{k}^{\;}-\frac{1}{\sigma_{b_{i}}^{2}},\\ \lambda_{ab_{i}}^{\;}=D^{2}e_{\;}^{2\ln\;a_{i}}\sum_{k=1}^{q}\left[\left(X_{k}-b_{i}\right)w_{ik}^{*}\times {\bar{f}}_{k}^{\;}\right], \end{array} \end{array}$$

where

(28) $$\begin{array}{l} w_{ik}^{*}=p_{i}^{*}\left(X_{k}\right)\times \left(1-p_{i}^{*}\left(X_{k}\right)\right), \end{array}$$

which lead to the Fisher-scoring algorithm for the BEMM:

(29) $$\begin{array}{l} \left[\begin{array}{c} \ln\;a_{i}^{\left(t+1\right)}\\ b_{i}^{\left(t+1\right)} \end{array}\right]=\left[\begin{array}{c} \ln\;a_{i}^{\left(t\right)}\\ b_{i}^{\left(t\right)} \end{array}\right]-{\left[\begin{array}{cc} \lambda_{aa_{i}}^{\;} & \lambda_{ab_{i}}^{\;}\\ \lambda_{ab_{i}}^{\;} & \lambda_{bb_{i}}^{\;} \end{array}\right]}^{-1}\left[\begin{array}{c} \lambda_{a_{i}}^{\;}\\ \lambda_{b_{i}}^{\;} \end{array}\right]. \end{array}$$

In this case, the estimation of the *c* parameter is separated from that of *a* and b, so the BEMM has a simplified 2-by-2 Hessian matrix (negative information matrix) in the iteration formulation.

**Figure 1:**
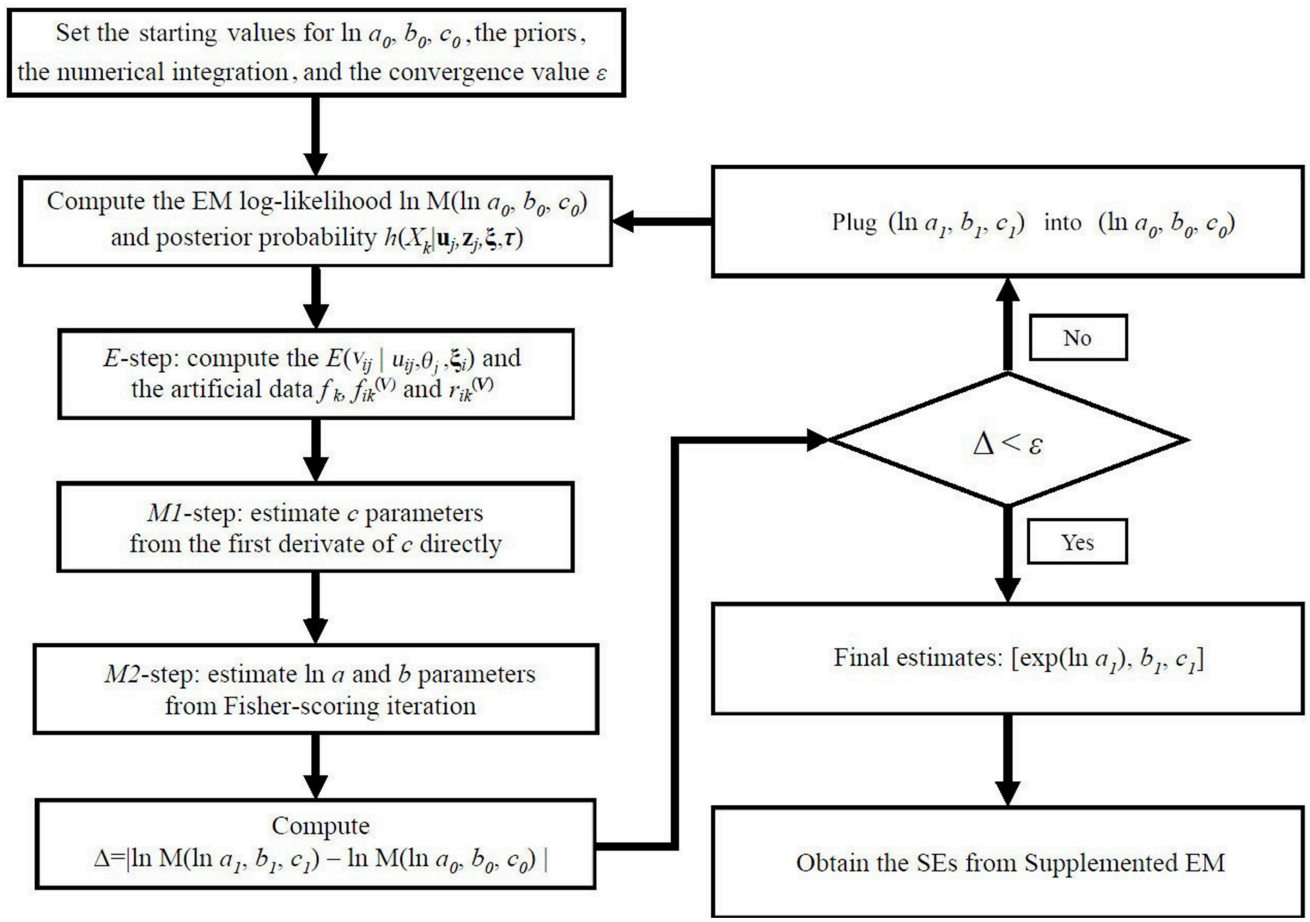
The flow chart of the BEMM.

To summarize, the flow chart of the BEMM has been given in Figure 1.

The same line of reasoning can be used to develop a BEMM for the second reformulation and the details are presented in Appendix A.

### Standard Errors (SEs) of Parameter Estimation

Both the BEMM and the Bayesian EM are members of the EM family and it is well recognized that one drawback of the EM algorithms is that estimation SEs are not the natural products of their implementation, so we still need a practical method to obtain SEs (McLachlan and Krishnan, 2007). In detail, SEs can be calculated via the Fisher information matrix (Thissen and Wainer, 1982), empirical cross-product approximation (Jones and Geoffrey, 1992), the supplemented EM (SEM) method (Meng and Rubin, 1991; Cai, 2008), the forward difference method and the Richardson extrapolation method (Jamshidian and Jennrich, 2000), or sandwich covariance matrix (Kauermann and Carroll, 2001). Recently, the SEM method has been extended to various IRT models (Cai, 2008; Cai and Lee, 2009; Tian et al., 2013) and proved to flexible enough to handle complex models in IRT. The current study, therefore, will focus on how to apply the SEM to the BEMM.

Cai and Lee (2009) has given a general SEM formulation for the large-sample covariance matrix as:

(30) $$\begin{array}{l} V\left({\hat{\xi }}_{i}|Y\right)=\Phi^{-1}\left({\hat{\xi }}_{i}|Y\right)=\Phi_{c}^{-1}\left({\hat{\xi }}_{i}|Y\right){\left\{E_{d}-\Delta \left({\hat{\xi }}_{i}\right)\right\}}^{-1}, \end{array}$$

where $\Phi^{-1}\left({\hat{\xi }}_{i}|Y\right)$ is the inverse of item information matrix, *E*_*d*_ is the identity matrix with 3 dimensions, and $\Delta \left({\hat{\xi }}_{i}\right)$ can be calculated from the Fisher-scoring execution. Please note that from Equation (10), in Bayesian approach (both Bayesian EM and Bayesian EMM), $\Phi^{-1}\left({\hat{\xi }}_{i}|Y\right)$ involves not only the likelihood component but also the prior component. Refer to Cai and Lee (2009) for additional details. It is worth noting that the likelihood function for the BEMM algorithms are different the one for the 3PLM due to mixture modeling reformulation, so the SEs for the BEMM are different from those for the BME.

Firstly, the item information matrix of the BEMM can be obtained from Equations (23, 27) as:

(31) $$\begin{array}{l} \Phi_{c}\left({\hat{\xi }}_{i}|Y\right)=\left[\begin{array}{ccc} -\lambda_{aa_{i}}^{\;} & -\lambda_{ab_{i}}^{\;} & 0\\ -\lambda_{ab_{i}}^{\;} & -\lambda_{bb_{i}}^{\;} & 0\\ 0 & 0 & -\lambda_{cc_{i}}^{\;} \end{array}\right]. \end{array}$$

Then, the Δ matrix was also simplified due to the covariance between *c* and (*a, b*) equaling 0:

(32) $$\begin{array}{l} \Delta \left({\hat{\xi }}_{i}\right)\text{=}\left[\begin{array}{ccc} \delta_{aa_{i}}^{\;} & \delta_{ab_{i}}^{\;} & 0\\ \delta_{ab_{i}}^{\;} & \delta_{bb_{i}}^{\;} & 0\\ 0 & 0 & \delta_{cc_{i}}^{\;} \end{array}\right]. \end{array}$$

Finally, SEs of the Bayesian EMM can be obtained from:

(33) $$\begin{array}{l} \left(SE_{a_{i}}^{\;},SE_{b_{i}}^{\;},SE_{c_{i}}^{\;}\right)=\sqrt{diag\left\{\Phi_{c}^{-1}\left({\hat{\xi }}_{i}|Y\right){\left[E_{3}-\Delta \left({\hat{\xi }}_{i}\right)\right]}^{-1}\right\}}. \end{array}$$

Obviously, the covariance elements between the guessing parameter and other two parameters are not zero in the traditional MLE/EM and BEM, but due to the setup of two maximization steps in EMM, these elements can be legitimately set to zero. The zero covariance removes undesirable fluctuation in the item parameter estimation and thus makes the estimated SEs smaller than the counterparts, especially the guessing parameter, than in the Bayesian EM.

## Simulation Study

The simulation study intends to demonstrate how different priors impact the Bayesian EMM algorithms, and compares it with the Bayesian EM in BILOG-MG under two different priors for *c* parameters. One prior in this study comes from BILOG-MG default setting, *c* ~ *Beta*(4, 16) with μ = 0.2, σ^2^ = 0.008, the other is a more non-informative prior from the flexMIRT (Houts and Cai, 2015) default setting, *c* ~ *Beta*(1, 4) with μ = 0.2, σ^2^ = 0.027.

To implement these algorithms, we developed a MATLAB toolbox, IRTEMM, to obtain the BEMM estimates. IRTEMM also offers several different options for estimating SEs including SEM.

### Data Generation

Following Mislevy (1986)'s setting for data generation, the current study simulated the item parameters *a, b, c* for 10 and 20 items from an independent normal distribution follows ln *a* ~ *N*(0, 0.5) where (0.3 ≤ *a* ≤ 2.5); *b* ~ *N*(0, 1)where (−3 ≤ *b* ≤ 3); andlogit *c* ~ *N*(−1.39, 0.16). Three sample sizes of examinees (1,000, 1,500 and 2,000) were simulated from the standard normal distribution. For each condition, we ran 50 replications for each condition in the fully crossed 4 (two BEMM methods vs. two BILOG-MG methods) × 3(1,000 vs. 1,500 vs. 2,000) × 2(10 vs. 20) design.

### Evaluation Criteria

The evaluation criteria for item parameter recovery are bias and the root mean squared error (RMSE), which are calculated as:

$$\begin{array}{l} bias=\frac{\sum_{s=1}^{S=50}\left({\hat{\psi }}_{is}-\psi_{i}\right)}{S},\;RMSE=\sqrt{\frac{\sum_{s=1}^{S=50}{\left({\hat{\psi }}_{is}-\psi_{i}\right)}^{2}}{S}}. \end{array}$$

Due to space constraint, the complete results have been summarized in Appendix C and only that for the condition of 1,000 examinees and 20 items here will be shown here since the others conditions presented a very similar pattern.

### Results

**Figure 2:**
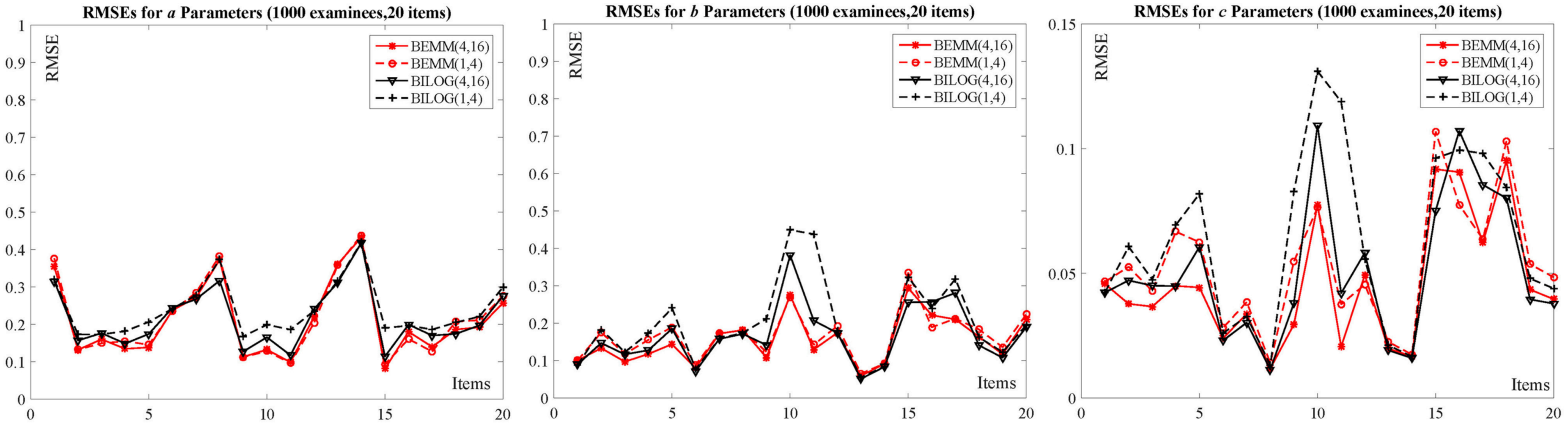
The RMSEs for 1000 examinees with 20 items.

As can be seen from Table 2: The biases across the four conditions for *a* and *b* parameters seem very similar, but for *c* parameters, these biases are highly influenced by priors. Both the absolute values of biases from the Bayesian EMM for *c* parameters are smaller than the Bayesian EM in BILOG-MG when changing priors. A more intuitive conclusion can be drawn from Figure 2: The RMSEs of *c* parameters from the right plot shows the Bayesian EMM has lower RMSE than the Bayesian EM in BILOG-MG. Furthermore, the difference of RMSEs produced by the Bayesian EMM between two prior conditions are much smaller than the Bayesian EM, which means the Bayesian EMM tends to be less affected by priors and yields more stable estimates than the Bayesian EM.

**Table 2:** The Biases for item parameter estimates with 1000 examinees and 20 items.

**Item**	**Generating**			**Biases for** ***a***				**Biases for** ***b***				**Biases for** ***c***			
		**Bayesian EMM**		**BILOG-MG**		**Bayesian EMM**		**BILOG-MG**		**Bayesian EMM**		**BILOG-MG**			
	***a***	***b***	***c***	***Beta*(4, 16)**	***Beta*(1, 4)**	***Beta*(4, 16)**	***Beta*(1, 4)**	***Beta*(4, 16)**	***Beta*(1, 4)**	***Beta*(4, 16)**	***Beta*(1, 4)**	***Beta*(4, 16)**	***Beta*(1, 4)**	***Beta*(4, 16)**	***Beta*(1, 4)**
1	1.547	0.540	0.292	0.051	0.092	0.051	0.089	−0.044	−0.033	−0.026	−0.012	−0.019	−0.012	−0.015	−0.008
2	0.605	0.705	0.182	0.089	0.074	0.120	0.128	−0.017	−0.055	0.045	0.041	0.009	−0.005	0.028	0.026
3	0.692	0.592	0.143	0.113	0.081	0.135	0.117	0.036	−0.015	0.077	0.045	0.023	0.003	0.036	0.023
4	0.958	−0.160	0.216	0.044	0.043	0.068	0.086	0.002	−0.012	0.042	0.051	−0.012	−0.019	0.004	0.007
5	0.581	0.393	0.189	0.089	0.082	0.127	0.148	0.016	−0.018	0.096	0.110	0.011	−0.001	0.035	0.040
6	1.477	0.909	0.151	0.131	0.101	0.144	0.121	−0.045	−0.053	−0.028	−0.035	−0.002	−0.007	0.001	−0.003
7	1.076	1.634	0.232	0.040	0.039	0.055	0.062	−0.132	−0.133	−0.109	−0.111	−0.018	−0.019	−0.014	−0.014
8	1.548	2.107	0.106	0.018	−0.028	−0.008	−0.028	−0.121	−0.116	−0.098	−0.099	−0.002	−0.005	−0.001	−0.004
9	0.911	−0.791	0.189	0.061	0.050	0.079	0.098	0.058	0.027	0.098	0.106	−0.006	−0.024	0.014	0.014
10	0.480	0.064	0.123	0.107	0.096	0.142	0.165	0.227	0.178	0.329	0.351	0.068	0.051	0.098	0.104
11	0.480	−0.547	0.195	0.071	0.065	0.090	0.144	0.055	0.015	0.153	0.331	−0.002	−0.016	0.029	0.081
12	0.509	1.533	0.126	0.182	0.148	0.207	0.192	−0.042	−0.077	0.002	−0.017	0.042	0.026	0.053	0.045
13	2.081	0.886	0.122	0.093	0.042	0.093	0.054	−0.036	−0.044	−0.020	−0.027	−0.001	−0.006	0.001	−0.003
14	2.234	1.458	0.198	−0.269	−0.276	−0.336	−0.335	−0.057	−0.057	−0.040	−0.042	−0.005	−0.006	−0.004	−0.005
15	0.651	0.229	0.294	−0.020	−0.017	0.036	0.107	−0.246	−0.254	−0.107	−0.001	−0.082	−0.085	−0.039	−0.006
16	0.872	−0.539	0.075	0.143	0.112	0.161	0.140	0.203	0.146	0.237	0.190	0.082	0.050	0.098	0.070
17	0.532	0.152	0.128	0.122	0.104	0.152	0.157	0.148	0.089	0.221	0.207	0.049	0.027	0.072	0.066
18	1.319	−0.277	0.310	−0.105	−0.080	−0.068	0.002	−0.119	−0.106	−0.076	−0.024	−0.084	−0.077	−0.063	−0.039
19	1.141	0.558	0.227	0.062	0.063	0.081	0.092	−0.048	−0.054	−0.024	−0.023	−0.017	−0.019	−0.010	−0.009
20	0.666	1.510	0.233	0.133	0.137	0.173	0.195	−0.123	−0.130	−0.078	−0.074	−0.003	−0.005	0.009	0.013

With the increasing of examinees from 1,000 to 2,000, the item parameter recovery of the Bayesian EMM and the Bayesian EM in BILOG-MG is obviously improved, and the difference between two priors for both methods is also decreased. However, some relatively large biases and RMSEs of the Bayesian EM still exist due to changing priors even in the largest sample size of the current study (e.g., 2,000 examinees and 20 items), while those from the Bayesian EMM are more stable (see Appendix C for details). In addition, the increase of the item number from 10 to 20 has no obvious influence for majority of item parameters.

Thus, it can be concluded that the Bayesian EMM inherits the advantages of both the EMM and the Bayesian method, and yields better estimates than the Bayesian EM. In other words, the Bayesian EMM has the most stable solutions among the two methods.

## Two Empirical Examples

**Figure 3:**
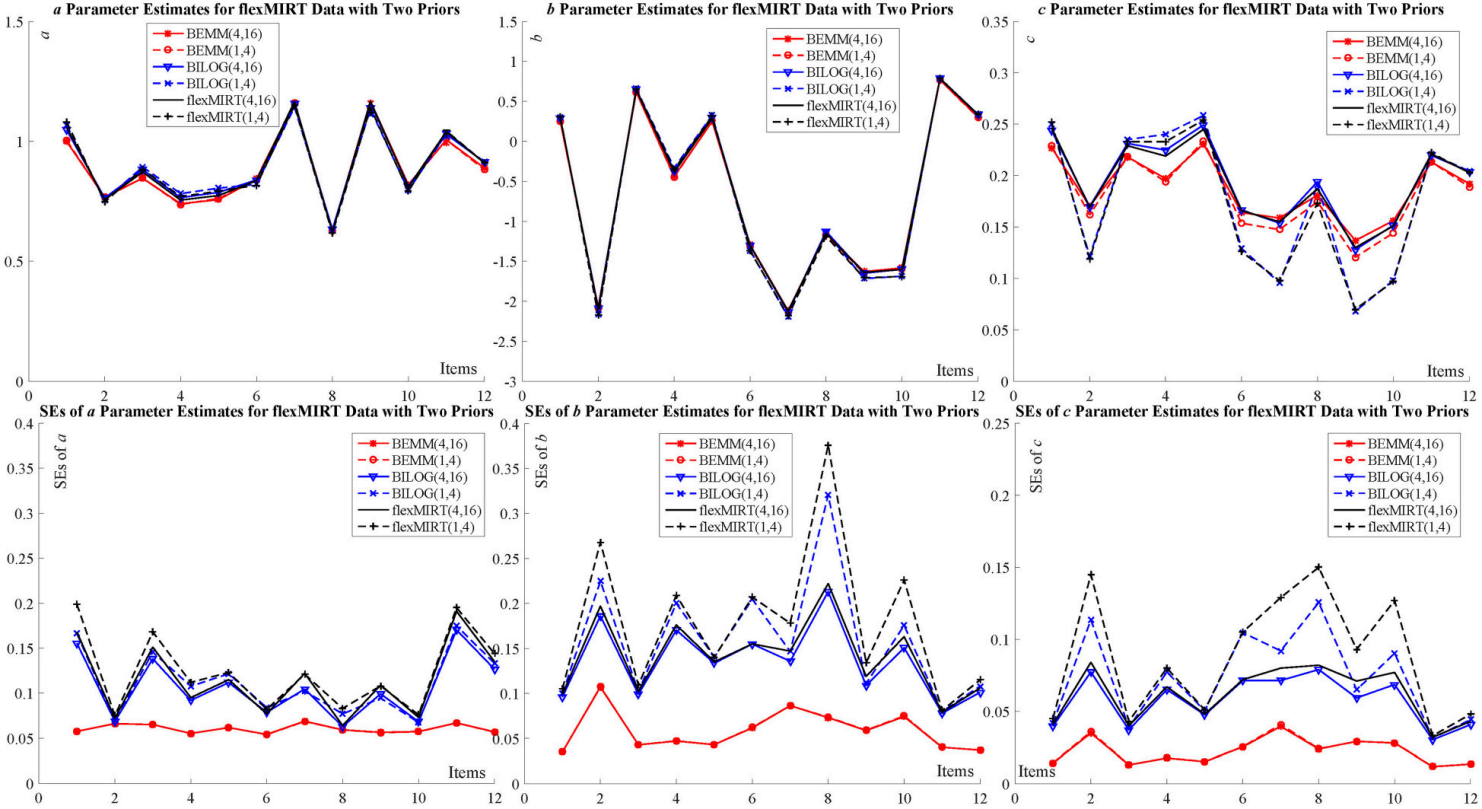
Item parameter calibration for flexMIRT data.

**Figure 4:**
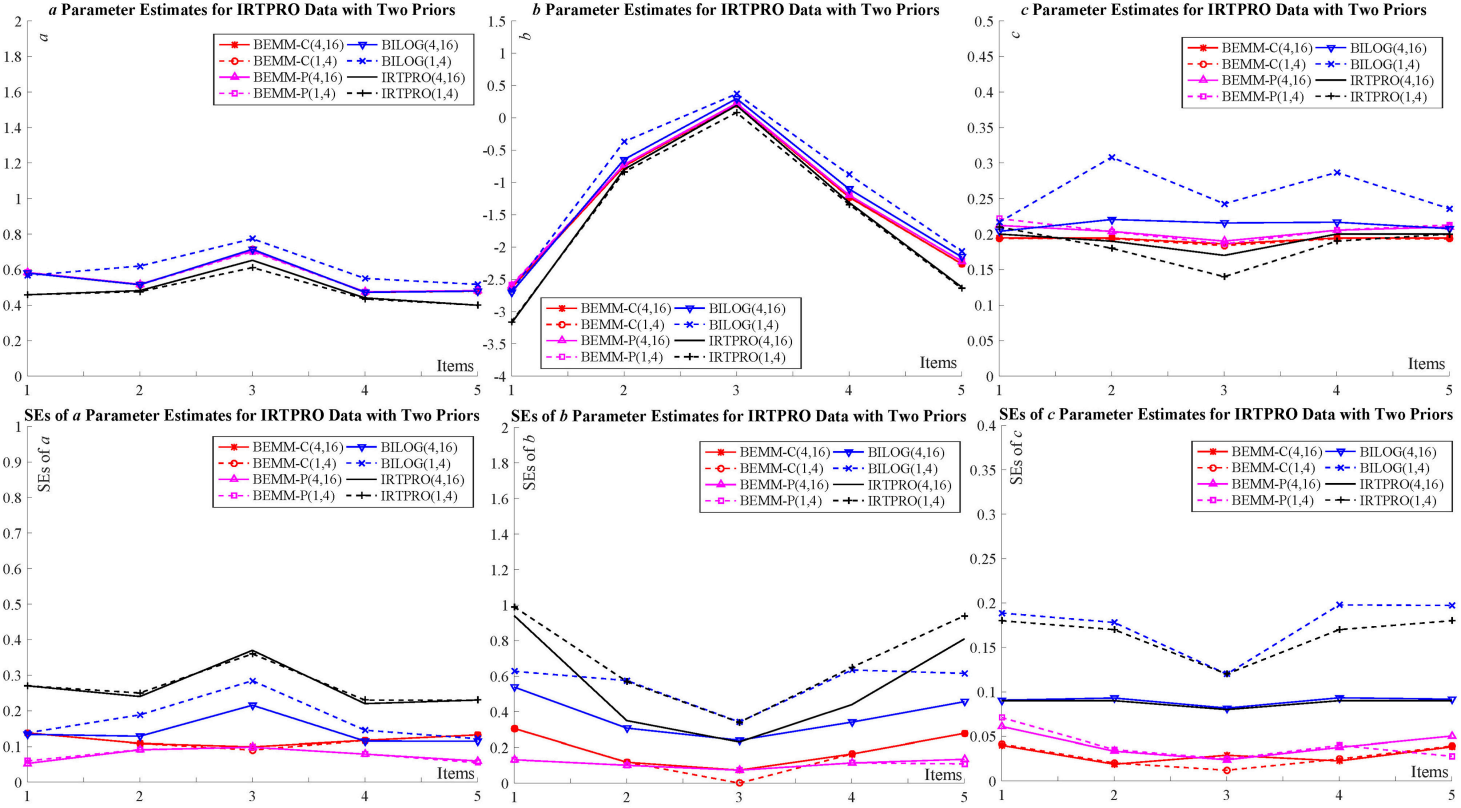
Item parameter calibration for IRTPRO data.

Two empirical examples of different sample sizes and item numbers are given here to demonstrate feasibility of the BEMM in practice: The first dataset represents a case where the numbers of items and examinees are relatively large while the other small. The estimates of the BEMM and the EMM can be obtained from the MATLAB toolbox, IRTEMM. As for the Bayesian EM, in addition to BILOG-MG, we also use two of the most recent IRT programs flexMIRT (Houts and Cai, 2015) and IRTPRO (Cai et al., 2011) to carry out a cross-implementation validation. The complete results of two examples are summarized in the tables and figures in Appendix D. Only two figures (Figure 3 for flexMIRT data and Figure 4 for IRTPRO data) are presented here to compare with the results of the BEMM and BILOG-MG analyses.

### The Dataset From flexMIRT

The first dataset is the flexMIRT example “g341-19.txt,” which consists of the responses to 12 items from 2,844 examinees. This example aimed at demonstrating whether the BEMM is more robust against the differential effects of priors than the Bayesian EM. The default settings for guessing priors in BILOG-MG and flexMIRT [*c* ~ *Beta* (4,16) vs. *c* ~ *Beta* (1,4)] have been applied to the BEMM and both software implementations.

The results are presented in Figure 3. The estimates for both *a* and *b* parameters are in each other's proximity for all different implementations. The estimates for *c* parameters present non-negligible divergence for different guessing priors for the BILOG-MG and flexMIRT: the estimates of these implementations cluster in two groups based on the guessing prior setting. The BEMM, in contrast, produced almost identical estimates under different prior setting. This cross-software validation shows that (1) the divergence in point estimates is possibly inherent in the Bayesian EM algorithm, but not due to different software executions; (2) the BEMM algorithm can provide stable point estimates that are robust against change in priors; and (3) correspondingly, there is noticeable difference in SEs for different priors for BILOG-MG and flexMIRT while there is no such difference for the BEMM.

### The Dataset From IRTPRO

The dataset of the second example is from the IRTPRO example “lsat6.csv,” which consists of responses to 5 items from 1,000 examinees. The dataset originally came from the Law School Admissions Test Section 6 (LSAT6) and has been widely used as an example in the item response theory (Bock and Lieberman, 1970; McDonald, 1999; Du Toit, 2003; Chalmers, 2012). More importantly, this dataset presents a case with realistically small number of examinees in educational testing scenarios and Bayesian EMM's performance with this dataset testify its applicability in practice. Following the IRTPRO default setting, the priors for the *a* and *b* parameters as: ln *a* ~ *N*(0, 1) and *b* ~ *N*(0, 3).

Figure 4 shows that, all of the item point estimates for *a* and *b* parameters among seven conditions which are very similar and relatively low (0.40 ≤ *a* ≤ 0.77; −3.18 ≤ *b* ≤ 0.37). In this adverse situation, the *c* parameters estimated by the Bayesian EM are obviously unstable and they are highly affected by priors. However, the Bayesian EMM estimates under two priors are comparatively accordant and very close to the MLE solutions. As regards SEs, the four lines of BILOG-MG and IRTPRO can be divided into two groups according to their priors, while there is no obvious difference in both results of the Bayesian EMM.

To summarize, these two examples illustrate that the Bayesian EMM was less affected by priors since they take the full advantage of the EMM and the Bayesian method.

## Discussion and Future Directions

Based on the results of the simulation study and real-world examples, the conclusions can be summarized as follows: (1) the BEMM can yield at least comparable or even better item estimates than the Bayesian EM; (2) the BEMM is less sensitive to change in item priors than the Bayesian EM, despite of the implementations; both point estimates and SEs, especially for the guessing parameters, are subjected to less fluctuation than BILOG-MG, flexMIRT and IRTPRO when different priors are used.

Obviously, the BEMM takes full advantage of the EMM and the Bayesian approach. On the one hand, the EMM itself is a more powerful MMLE method than the EM, so the BEMM can explore the likelihood function as thoroughly as the EMM before turning to priors to “shrink” the estimates; on the other hand, the Bayesian approach can naturally be used to solve the issue of estimate inflation for some troublesome items even when the EMM cannot produce reasonable MLE estimates. The simulation study and the two real dataset examples are of limited scopes, so the conclusions based on their results should be interpreted with caution. It is not the intent of this paper to advocate for the elimination of the usage of other methods. The BEMM can be used to check with the Bayesian EM in the IRT programs in practice. Due to the high complexity in real-world 3PLM data, a combination of the Bayesian EM in different implementations, the BEMM and even the naked MLE solution, the EMM, might lead to a more sophisticated and nuanced understanding of data.

Several questions deserve further attention. Firstly, the BEMM can be readily extended to other IRT models with guessing effect. A case in point is the IRT model with covariates model (Tay et al., 2013). According to Tay et al. (2016), it needs at least a sample of 20,000 examinees to fit a 3PLM with covariates successfully. In this case, the BEMM may offer a better alternative to reducing the required sample sizes for the 3PLM with covariates.

Secondly, the mixture modeling approach and the BEMM can be naturally accommodated for the 4PLM (Barton and Lord, 1981). There is a renewed interest in the 4PLM (Rulison and Loken, 2009; Loken and Rulison, 2010; Liao et al., 2012; Ogasawara, 2012; Feuerstahler and Waller, 2014; Culpepper, 2015) for its usefulness in achievement test and psychological datasets. But, the estimation challenge posed by the 4PLM is greater than the 3PLM due to the additional slipping parameter in the model (Loken and Rulison, 2010; Culpepper, 2015, 2017; Waller and Feuerstahler, 2017). Adaption of the BEMM for the 4PLM is a promising direction.

## Author Contributions

SG: software and implementation, mathematical derivation, simulation studies and examples, responding, writing—revision, writing—review, editing, methodology, data curation, and validation. CZ: formal analysis, methodology, conceptualization, funding acquisition, investigation, software, project administration, resources, supervision, writing—original draft, writing—review, and editing.

### Conflict of Interest Statement

The authors declare that the research was conducted in the absence of any commercial or financial relationships that could be construed as a potential conflict of interest.
