# Supplementary material for: The Bayesian Expectation-Maximization-Maximization for the 3PLM
Source: Front Psychol. 2019 May 31;10:1175. doi: 10.3389/fpsyg.2019.01175 (PMC6555200; doi:10.3389/fpsyg.2019.01175)
Supplement: Supplementary file 1 [file Table_1.DOCX]

Supplementary Material

The Bayesian Expectation-Maximization-Maximization for the 3PLM

Shaoyang Guo, Chanjin Zheng *

*** Correspondence:** Chanjin Zheng: [russelzheng@gmail.com](mailto:russelzheng@gmail.com)

The supplementary materials include four Appendixes.

**The Appendix A** provides the details of the EMM (g-process) algorithm proposed by Zheng et al. (2017) ………………………………………………………………………………………. Page 2

**The Appendix B** provides the full details of the derivation from Equation 17, and all of the math notations is the same as in the article ………………………………………………...……. Page 15

**The Appendix C** provides the complete results (RMSE & bias) of simulation study. .….. Page 20

**The Appendix D** provides the complete results of two real-world examples …………...... Page 33

# Appendix A

The 3PLM can be conceived as an extension of the 2PLM with an item-specific guessing parameter:

$$P_{i}(\theta_{j})=c_{i}\times\left[1\right]+(1-c_{i})\times\left[P_{i}^{*}(\theta_{j})\right],$$

with

$$P_{i}^{*}(\theta_{j})=\frac{1}{1+\exp\left(-Da_{i}(\theta_{j}-b_{i})\right)}.$$

as the 2PLM.

According to Zheng, Meng, Guo, and Liu (2017), a latent indicator variable $z_{ij}$ is introduced to indicate whether the guessing strategy is employed by an examinee:

$$z_{ij}=\left\{\begin{matrix} 1\text{ if examinee }j\text{ does not guess on item }i\text{;} \\ 0\text{ if examinee }j\text{ does guess on item }i\text{. } \end{matrix}\right.$$

Reasonably, $z_{ij}$ follows a Bernoulli distribution with parameter $1-c_{i}$, or $P\left(z_{ij}=1\right)=1-c_{i}$. To simplify the derivation in the sequel, $z_{ij}$ is always assumed to be conditionally independent of $\theta_{j}$ given the response $u_{ij}$, which means that, for each item, the examinee decides randomly whether guessing or ability-based responding is chosen first (San Martín et al., 2006; von Davier, 2009b). From the mixture-modeling perspective, depending on the value of $z_{ij}$, the possibilities of 3PLM can be decomposed into two irrelevant parts: 1 and $P_{i}^{*}(\theta_{j})$.

Furthermore, the conditional possibilities can be easily obtained as:

$$\begin{matrix} P(u_{ij}=1|z_{ij}=1,\theta_{j},\mathbf{\xi}_{i})=P_{i}^{*}(\theta_{j}),\text{ }P(u_{ij}=1|z_{ij}=0,\theta_{j},\mathbf{\xi}_{i})=1, \\ P(u_{ij}=0|z_{ij}=1,\theta_{j},\mathbf{\xi}_{i})=1-P_{i}^{*}(\theta_{j}),\text{ }P(u_{ij}=0|z_{ij}=0,\theta_{j},\mathbf{\xi}_{i})=0. \end{matrix}$$

By the multiplication rule, , the joint distribution of $u_{ij}$ and $z_{ij}$ can be calculated as:

$$\begin{matrix} P(u_{ij}=1,z_{ij}=1|\theta_{j},\mathbf{\xi}_{i})=(1-c_{i})P_{i}^{*}(\theta_{j}),\text{ }P(u_{ij}=1,z_{ij}=0|\theta_{j},\mathbf{\xi}_{i})=c_{i}, \\ P(u_{ij}=0,z_{ij}=1|\theta_{j},\mathbf{\xi}_{i})=(1-c_{i})(1-P_{i}^{*}(\theta_{j})),\text{ }P(u_{ij}=0,z_{ij}=0|\theta_{j},\mathbf{\xi}_{i})=0. \end{matrix}$$

Note that $(1-c_{i})P_{i}^{*}(\theta_{j})+c_{i}+(1-c_{i})(1-P_{i}^{*}(\theta_{j}))=1$, so $P(u_{ij}=0,z_{ij}=0|\theta_{j},\mathbf{\xi}_{i})=0$ is actually redundant and can be omitted from the probability density function.

Take the first reformulation as an example to illustrate how to derive the BEMM algorithm. Following Mislevy (1986)'s parameterization to take logarithmic form of $a_{i}$, the 3PLM can be rewritten as:

$$p_{i}(\theta_{j})=c_{i}+(1-c_{i})\times p_{i}^{*}(\theta_{j}),$$

with

$$p_{i}^{*}(\theta_{j})=\frac{1}{1+\exp\left(-De^{\ln a_{i}}(\theta_{j}-b_{i})\right)}.$$

Mislevy (1986) also has given a general Bayesian formulation for the 3PLM which we may apply to the BEMM as well. Let $\psi_{i}$ represents any item parameter for item $i$ in $\mathbf{\xi}_{i}$, and then the first derivative of the general Bayesian formulation for each item parameter can be obtained as:

$$\frac{\partial\ln L(\mathbf{U},Z|\mathbf{\xi})}{\partial\psi_{i}}+\frac{\partial\ln g(\psi_{i}|\eta)}{\partial\psi_{i}},$$

where $L(\mathbf{U},Z|\mathbf{\xi})$ is the likelihood of the EMM with the logarithmic form of $a_{i}$, and $g(\psi_{i}|\eta)$ is the item parameter prior distribution for item $i$, $\mathbf{U}$ is the $n\times N$ response matrix with $u_{ij}$ as its elements; $\mathbf{Z}$ is defined, with respect to $z_{ij}$, in analogy to $\mathbf{U}$ and $u_{ij}$; $\mathbf{\xi}=\left(\xi_{1},\xi_{2},...,\xi_{i},...,\xi_{n}\right)$ is the matrix for item parameters; $g\left(\theta_{j}|\mathbf{\tau}\right)$ is a density function for examinees’ ability, and $\mathbf{\tau}$ is the vector containing the parameters of the examinee population ability distribution.

In detail, based on Equation A.4, the joint probability can be obtained as:

$$P(\mathbf{u}_{j},\mathbf{z}_{j}|\theta_{j},\mathbf{\xi})=\prod_{i=1}^{n} [(1-c_{i})p_{i}^{*}(\theta_{j})]^{u_{ij}z_{ij}}\times c_{i}^{u_{ij}(1-z_{ij})}\times[(1-c_{i})\left(1-p_{i}^{*}(\theta_{j})\right)]^{(1-u_{ij})z_{ij}}.$$

Furthermore, the joint distribution for the new augmented complete data $\left(\mathbf{U},\mathbf{Z},\mathbf{\theta}\right)$ in which $\mathbf{\theta}=\left(\theta_{1},...\theta_{j},...\theta_{N}\right)$will be

$$P(\mathbf{u}_{j},\mathbf{z}_{j},\theta_{j}|\mathbf{\xi},\mathbf{\tau})=P(\mathbf{u}_{j},\mathbf{z}_{j}|\theta_{j},\mathbf{\xi})g(\theta_{j}|\mathbf{\tau}).$$

By integrating out the ability variable, the marginal distribution for a single examinee $j$ is

$$P(\mathbf{u}_{j},\mathbf{z}_{j}|\mathbf{\xi})=\int_{\theta_{j}} P(\mathbf{u}_{j},\mathbf{z}_{j}|\mathbf{\xi},\theta_{j})g(\theta_{j}|\mathbf{\tau})d\theta_{j}.$$

Thus, the likelihood function in Bayesian EMM can be calculated from

$$\begin{matrix} L\equiv L(\mathbf{U},Z|\mathbf{\xi})=\prod_{j=1}^{N} P(\mathbf{u}_{j},\mathbf{z}_{j}|\mathbf{\xi}) \\ \text{ }=\prod_{j=1}^{N} \int_{\theta_{j}} P(\mathbf{u}_{j},\mathbf{z}_{j}|\mathbf{\xi},\theta_{j})g(\theta_{j}|\mathbf{\tau})d\theta_{j} \\ \text{ }=\prod_{j=1}^{N} \int_{\theta_{j}} \left(\prod_{i=1}^{n} [(1-c_{i})p_{i}^{*}(\theta_{j})]^{u_{ij}z_{ij}}\times c_{i}^{u_{ij}(1-z_{ij})}\times[(1-c_{i})\left(1-p_{i}^{*}(\theta_{j})\right)]^{(1-u_{ij})z_{ij}}\right)g(\theta_{j}|\mathbf{\tau})d\theta_{j}. \end{matrix}$$

Then, the first derivative of item parameters from log-likelihood function can be derived as

$$\begin{matrix} \frac{\partial\ln L}{\partial\psi_{i}}=\sum_{j=1}^{N} \frac{\partial\ln P(\mathbf{u}_{j},\mathbf{z}_{j}|\mathbf{\xi}_{i})}{\partial\psi_{i}} \\ \text{ }=\sum_{j=1}^{N} \frac{1}{P(\mathbf{u}_{j},\mathbf{z}_{j}|\mathbf{\xi}_{i})}\frac{\partial P(\mathbf{u}_{j},\mathbf{z}_{j}|\mathbf{\xi}_{i})}{\partial\psi_{i}} \\ \text{ }=\sum_{j=1}^{N} \frac{1}{P(\mathbf{u}_{j},\mathbf{z}_{j}|\mathbf{\xi}_{i})}\int_{\theta_{j}} \frac{\partial P(\mathbf{u}_{j},\mathbf{z}_{j}|\theta_{j},\mathbf{\xi}_{i})g(\theta_{j}|\mathbf{\tau})d\theta_{j}}{\partial\psi_{i}} \\ \text{ }=\sum_{j=1}^{N} \frac{1}{P(\mathbf{u}_{j},\mathbf{z}_{j}|\mathbf{\xi}_{i})}\int_{\theta_{j}} \left[\frac{\partial\ln P(\mathbf{u}_{j},\mathbf{z}_{j}|\theta_{j},\mathbf{\xi}_{i})}{\partial\psi_{i}}\right]P(\mathbf{u}_{j},\mathbf{z}_{j}|\theta_{j},\mathbf{\xi}_{i})g(\theta_{j}|\mathbf{\tau})d\theta_{j} \\ \text{ }=\sum_{j=1}^{N} \int_{\theta_{j}} \left[\frac{\partial\ln P(\mathbf{u}_{j},\mathbf{z}_{j}|\theta_{j},\mathbf{\xi}_{i})}{\partial\psi_{i}}\right]\left[\frac{P(\mathbf{u}_{j},\mathbf{z}_{j}|\theta_{j},\mathbf{\xi}_{i})g(\theta_{j}|\mathbf{\tau})}{P(\mathbf{u}_{j},\mathbf{z}_{j}|\mathbf{\xi}_{i})}\right]d\theta_{j} \end{matrix}$$

$$\begin{matrix} \text{Let }R_{h}\text{=}[(1-c_{h})p_{h}^{*}(\theta_{j})]^{u_{hj}z_{hj}}\times c_{h}^{u_{hj}(1-z_{hj})}\times[(1-c_{h})\left(1-p_{h}^{*}(\theta_{j})\right)]^{(1-u_{hj})z_{hj}},\text{ } \\ \text{and }R_{i}\text{=}[(1-c_{i})p_{i}^{*}(\theta_{j})]^{u_{ij}z_{ij}}\times c_{i}^{u_{ij}(1-z_{ij})}\times[(1-c_{i})\left(1-p_{i}^{*}(\theta_{j})\right)]^{(1-u_{ij})z_{ij}}\text{, so we have} \\ \frac{\partial\ln L}{\partial\psi_{i}}=\sum_{j=1}^{N} \int_{\theta_{j}} \frac{\frac{\partial}{\partial\psi_{i}}\left[R_{i}\times\prod_{h\neq i}^{n} R_{h}\right]}{R_{i}\times\prod_{h\neq i}^{n} R_{h}}P\left(\theta_{j}\text{|}\mathbf{u}_{j},\mathbf{z}_{j},\mathbf{\tau},\mathbf{\xi}\right)d\theta_{j} \\ \text{ =}\sum_{j=1}^{N} \int_{\theta_{j}} \frac{\left[\frac{\partial R_{i}}{\partial\psi_{i}}\right]\times\prod_{h\neq i}^{n} R_{h}+\left[R_{i}\times\frac{\partial}{\partial\psi_{i}}\prod_{h\neq i}^{n} R_{h}\right]}{R_{i}\times\prod_{h\neq i}^{n} R_{h}}P\left(\theta_{j}\text{|}\mathbf{u}_{j},\mathbf{z}_{j},\mathbf{\tau},\mathbf{\xi}\right)d\theta_{j}\text{ } \\ \text{ =}\sum_{j=1}^{N} \int_{\theta_{j}} \left[\frac{1}{R_{i}\times\prod_{h\neq i}^{n} R_{h}}\left(\left[\frac{\partial R_{i}}{\partial\psi_{i}}\right]\times\prod_{h\neq i}^{n} R_{h}+R_{i}\times0\right)\right]P\left(\theta_{j}\text{|}\mathbf{u}_{j},\mathbf{z}_{j},\mathbf{\tau},\mathbf{\xi}\right)d\theta_{j} \\ \text{ }=\sum_{j=1}^{N} \int_{\theta_{j}} \left[\frac{1}{R_{i}}\frac{\partial R_{i}}{\partial\psi_{i}}\right]P\left(\theta_{j}\text{|}\mathbf{u}_{j},\mathbf{z}_{j},\mathbf{\tau},\mathbf{\xi}\right)d\theta_{j} \\ \text{ }=\sum_{j=1}^{N} \int_{\theta_{j}} \left[\frac{1}{R_{i}}\frac{\partial[(1-c_{i})p_{i}^{*}(\theta_{j})]^{u_{ij}z_{ij}}\times c_{i}^{u_{ij}(1-z_{ij})}\times[(1-c_{i})\left(1-p_{i}^{*}(\theta_{j})\right)]^{(1-u_{ij})z_{ij}}}{\partial\psi_{i}}\right]P\left(\theta_{j}\text{|}\mathbf{u}_{j},\mathbf{z}_{j},\mathbf{\tau},\mathbf{\xi}\right)d\theta_{j} \end{matrix}$$

$$=\sum_{j=1}^{N} \int_{\theta_{j}} \left[\left(\begin{matrix} \frac{\partial(1-c_{i})p_{i}^{*}(\theta_{j})}{\partial\psi_{i}}u_{ij}z_{ij}[(1-c_{i})p_{i}^{*}(\theta_{j})]^{u_{ij}z_{ij}-1}\times c_{i}^{u_{ij}(1-z_{ij})}\times[(1-c_{i})\left(1-p_{i}^{*}(\theta_{j})\right)]^{(1-u_{ij})z_{ij}} \\ +[(1-c_{i})p_{i}^{*}(\theta_{j})]^{u_{ij}z_{ij}}\times\frac{\partial c_{i}}{\partial\psi_{i}}u_{ij}(1-z_{ij})c_{i}^{u_{ij}(1-z_{ij})-1}\times[(1-c_{i})\left(1-p_{i}^{*}(\theta_{j})\right)]^{(1-u_{ij})z_{ij}} \\ +[(1-c_{i})p_{i}^{*}(\theta_{j})]^{u_{ij}z_{ij}}\times c_{i}^{u_{ij}(1-z_{ij})} \\ \text{ }\times\frac{\partial(1-c_{i})\left(1-p_{i}^{*}(\theta_{j})\right)}{\partial\psi_{i}}(1-u_{ij})z_{ij}[(1-c_{i})\left(1-p_{i}^{*}(\theta_{j})\right)]^{(1-u_{ij})z_{ij}-1} \end{matrix}\right)\frac{P\left(\theta_{j}\text{|}\mathbf{u}_{j},\mathbf{z}_{j},\mathbf{\tau},\mathbf{\xi}\right)}{R_{i}}\right]d\theta_{j}$$

where

$$\begin{matrix} P\left(\theta_{j}\text{|}\mathbf{u}_{j},\mathbf{z}_{j},\mathbf{\tau},\mathbf{\xi}\right)=P\left(\theta_{j}\text{|}\mathbf{u}_{j},\mathbf{\tau},\mathbf{\xi}\right)=\frac{P(\mathbf{u}_{j}|\theta_{j},\mathbf{\xi})g(\theta_{j}|\mathbf{\tau})}{\int_{\theta_{j}} P(\mathbf{u}_{j}|\theta_{j},\mathbf{\xi})g(\theta_{j}|\mathbf{\tau})d\theta_{j}}, \\ P(\mathbf{u}_{j}|\theta_{j},\mathbf{\xi})=\prod_{i=1}^{n} p_{i}(\theta_{j})^{u_{ij}}\times(1-p_{i}(\theta_{j}))^{1-u_{ij}}, \end{matrix}$$

Mislevy (1986) suggested that $\ln a_{i}\text{ and }b_{i}$ follow a normal distribution and $c_{i}$ a beta distribution, specifically,

$$\ln a_{i}\sim N(\mu_{\text{ln }a_{i}},\sigma_{\text{ln }a_{i}}^{2}),\text{ }b_{i}\sim N(\mu_{b_{i}},\sigma_{b_{i}}^{2}),\text{ }c_{i}\sim Beta(\alpha_{i},\beta_{i})$$

in which $\mu_{\text{ln }a_{i}},\mu_{b_{i}},\sigma_{\text{ln }a_{i}}^{2},\text{ and }\sigma_{b_{i}}^{2}$ are the means and variances for the corresponding normal distribution and $\alpha_{i},\beta_{i}$ are the parameters for the beta distribution for the guessing parameter. They may be specified as in the BILOG-MG default setting (Du Toit, 2003):

$$\ln a\sim N(0,0.5^{2}),b\sim N(0,2^{2}),c\sim Beta(4,16).$$

The first and second derivatives for the three priors are given by Mislevy (1986) as:

$$\begin{matrix} \frac{\partial\ln g(\text{ln }a_{i}|\mu_{\text{ln }a_{i}},\sigma_{\text{ln }a_{i}}^{2})}{\partial\text{ln }a_{i}}=-\frac{\ln a_{i}-\mu_{\text{ln }a_{i}}}{\sigma_{\text{ln }a_{i}}^{2}},\text{ }\frac{\partial^{2}\ln g(\text{ln }a_{i}|\mu_{\text{ln }a_{i}},\sigma_{\text{ln }a_{i}}^{2})}{\partial\text{ln }a_{i}\partial\text{ln }a_{i}}=-\frac{1}{\sigma_{\text{ln }a_{i}}^{2}}, \\ \frac{\partial\ln g(b_{i}|\mu_{b_{i}},\sigma_{b_{i}}^{2})}{\partial b_{i}}=-\frac{b_{i}-\mu_{b_{i}}}{\sigma_{b_{i}}^{2}},\text{ }\frac{\partial^{2}\ln g(b_{i}|\mu_{b_{i}},\sigma_{b_{i}}^{2})}{\partial b_{i}\partial b_{i}}=-\frac{1}{\sigma_{b_{i}}^{2}}, \\ \frac{\partial\ln g(c_{i}|\alpha_{i},\beta_{i})}{\partial c_{i}}=\frac{\alpha_{i}-1}{c_{i}}-\frac{\beta_{i}-1}{1-c_{i}}, \text{ }\frac{\partial^{2}\ln g(c_{i}|\alpha_{i},\beta_{i})}{\partial c_{i}\partial c_{i}}=-\frac{\alpha_{i}-1}{c_{i}^{2}}-\frac{\beta_{i}-1}{(1-c_{i})^{2}}. \end{matrix}$$

With the prior distribution component explained, the next will proceed to describe the Bayesian EMM method in which the likelihood component will be carefully delineated.

**Expectation Step and Artificial Data**

The expectation step boils down to calculation of the conditional expectations of $\mathbf{Z}$ and $\mathbf{\theta}$. From the joint distribution in Equation A.4, one can calculate the expectation of $z_{ij}$ conditional on $u_{ij}$ and the marginal distribution of $z_{ij}$. By the Bayesian rule,

$$\begin{matrix} P(z_{ij}=1|u_{ij}=1,\theta_{j},\mathbf{\xi}_{i})=\frac{(1-c_{i})p_{i}^{*}(\theta_{j})}{p_{i}(\theta_{j})}, \\ P(z_{ij}=1|u_{ij}=0,\theta_{j},\mathbf{\xi}_{i})=1. \end{matrix}$$

can be yielded from Equation A.3 and A.4. Then, the conditional expectation of $z_{ij}$ is

$$E(z_{ij}|u_{ij},\theta_{j},\mathbf{\xi}_{i})=\frac{(1-c_{i})p_{i}^{*}(\theta_{j})}{p_{i}(\theta_{j})}\times u_{ij}+1\times(1-u_{ij}).$$

As for $\mathbf{\theta}$, by using summation over a fixed grid of equally-spaced quadrature points $X_{k}\left(k=1,2,...,q\right)$ with an associated weight $A\left(X_{k}\right)$ to approximate integration, one can have the quadrature form of the first derivative of the expected log-likelihood function in Equation for each item parameter:

$$\frac{\partial\ln E\left[L\right]}{\partial\psi_{i}}\approx\sum_{j=1}^{N} \sum_{k=1}^{q} \left[\begin{matrix} \frac{u_{ij}E(z_{ij}|u_{ij},X_{k},\mathbf{\xi}_{i})}{p_{i}^{*}(X_{k})}\frac{\partial p_{i}^{*}(X_{k})}{\partial\psi_{i}}-\frac{(1-u_{ij})E(z_{ij}|u_{ij},X_{k},\mathbf{\xi}_{i})}{1-p_{i}^{*}(X_{k})}\frac{\partial p_{i}^{*}(X_{k})}{\partial\psi_{i}} \\ +\frac{u_{ij}(1-E(z_{ij}|u_{ij},X_{k},\mathbf{\xi}_{i}))}{c_{i}}\frac{\partial c_{i}}{\partial\psi_{i}}-\frac{E(z_{ij}|u_{ij},X_{k},\mathbf{\xi}_{i})}{1-c_{i}}\frac{\partial c_{i}}{\partial\psi_{i}} \end{matrix}\right]P\left(X_{k}\text{|}\mathbf{u}_{j},\mathbf{z}_{j},\mathbf{\tau},\mathbf{\xi}\right)$$

with

$$\begin{matrix} P\left(X_{k}\text{|}\mathbf{u}_{j},\mathbf{z}_{j},\mathbf{\tau},\mathbf{\xi}\right)=P\left(X_{k}\text{|}\mathbf{u}_{j},\mathbf{\tau},\mathbf{\xi}\right)\text{=}\frac{P(\mathbf{u}_{j}|X_{k},\mathbf{\xi})\times A\left(X_{k}\right)}{\sum_{k=1}^{q} P(\mathbf{u}_{j}|X_{k},\mathbf{\xi})\times A\left(X_{k}\right)}, \\ P(\mathbf{u}_{j}|X_{k},\mathbf{\xi})=\prod_{i=1}^{n} p_{i}(X_{k})^{u_{ij}}\times(1-p_{i}(X_{k}))^{1-u_{ij}}, \end{matrix}$$

where $P\left(X_{k}\text{|}\mathbf{u}_{j},\mathbf{z}_{j},\mathbf{\tau},\mathbf{\xi}\right)$ is the posterior probability of $\theta_{j}$ evaluated at $X_{k}$ given $\mathbf{u}_{j},\mathbf{z}_{j},\mathbf{\tau},\text{ and }\mathbf{\xi}$. To simplify the derivation, $X_{k}$ is assumed to be conditionally independent of $\mathbf{z}_{j}$given $\mathbf{u}_{j}$, then, $P\left(X_{k}\text{|}\mathbf{u}_{j},\mathbf{z}_{j},\mathbf{\tau},\mathbf{\xi}\right)$ equals $P\left(X_{k}\text{|}\mathbf{u}_{j},\mathbf{\tau},\mathbf{\xi}\right)$. Furthermore, $P\left(X_{k}\text{|}\mathbf{u}_{j},\mathbf{z}_{j},\mathbf{\tau},\mathbf{\xi}\right)$ can be used to compute the "artificial data". For instance, Bock and Aitkin (1981) has provided two fundamental artificial data for traditional EM algorithm as:

$$\begin{matrix} \bar{f}_{k}=\sum_{j=1}^{N} P\left(X_{k}\text{|}\mathbf{u}_{j},\mathbf{\tau},\mathbf{\xi}\right)=\sum_{j=1}^{N} P\left(X_{k}\text{|}\mathbf{u}_{j},\mathbf{z}_{j},\mathbf{\tau},\mathbf{\xi}\right), \\ \bar{r}_{ik}=\sum_{j=1}^{N} u_{ij}\times P\left(X_{k}\text{|}\mathbf{u}_{j},\mathbf{\tau},\mathbf{\xi}\right)=\sum_{j=1}^{N} u_{ij}\times P\left(X_{k}\text{|}\mathbf{u}_{j},\mathbf{z}_{j},\mathbf{\tau},\mathbf{\xi}\right), \end{matrix}$$

in which $\bar{f}_{k}$ is the expected number of examinees with ability $X_{k}$. Thus, the sum of $\bar{f}_{k}$ for every ability point $X_{k}$ equals the total number of examinees *N*. $\bar{r}_{ik}$ is the expected number of examinees with ability $X_{k}$ answering item $i$ correctly.

Then, the EMM algorithm introduced a new latent variable $\mathbf{Z}$, so there are two new artificial data as

$$\begin{matrix} \bar{f}_{ik}^{(Z)}=\sum_{j=1}^{N} E(z_{ij}|u_{ij},X_{k},\mathbf{\xi})P\left(X_{k}\text{|}\mathbf{u}_{j},\mathbf{z}_{j},\mathbf{\tau},\mathbf{\xi}\right), \\ \bar{r}_{ik}^{(Z)}=\sum_{j=1}^{N} u_{ij}\times E(z_{ij}|u_{ij},X_{k},\mathbf{\xi})P\left(X_{k}\text{|}\mathbf{u}_{j},\mathbf{z}_{j},\mathbf{\tau},\mathbf{\xi}\right), \end{matrix}$$

in which $\bar{f}_{ik}^{(Z)}$ is the expected number of examinees with ability $X_{k}$ without using guessing strategy; $\bar{r}_{ik}^{(Z)}$ is the expected number of examinees with ability $X_{k}$ answering item $i$correctly without using a guessing strategy. Thus, $\bar{r}_{ik}-\bar{r}_{ik}^{(Z)}$ is the expected number of examinees with ability $X_{k}$who can answer item $i$ correctly using the guessing strategy. The expected number of examinees with ability $X_{k}$ who can answer item $i$incorrectly by using the guessing strategy is zero, and this can be inferred from $P(u_{ij}=0,z_{ij}=0|\theta_{j},\mathbf{\xi}_{i})=0$. Putting these facts together, we know $\sum_{k=1}^{q} \left(\bar{r}_{ik}-\bar{r}_{ik}^{(Z)}+\bar{f}_{ik}^{(Z)}\right)$ is equivalent to the total number of examinees *N*, namely,`

$$\sum_{k=1}^{q} \left(\bar{r}_{ik}-\bar{r}_{ik}^{(Z)}+\bar{f}_{ik}^{(Z)}\right)=\sum_{k=1}^{q} \bar{f}_{k}=N.$$

After the E-step and calculation of the artificial data, the next steps are to compute the first and second derivatives of Equation A.19 with respect to each item parameter.

**Maximization Step-1 for *c* Parameters**

From Equation A.7 and Equation A.19, the first derivative for the guessing parameter is

$$\begin{matrix} \lambda_{c_{i}}=\frac{\partial\ln E\left[L\right]}{\partial c_{i}}+\frac{\partial\ln g(c_{i}|\alpha_{i},\beta_{i})}{\partial c_{i}} \\ \text{ }\approx\frac{\alpha_{i}-1}{c_{i}}-\frac{\beta_{i}-1}{1-c_{i}}+\sum_{j=1}^{N} \sum_{k=1}^{q} \left[\frac{u_{ij}(1-E(z_{ij}|u_{ij},X_{k},\mathbf{\xi}))}{c_{i}}-\frac{E(z_{ij}|u_{ij},X_{k},\mathbf{\xi})}{1-c_{i}}\right]P\left(X_{k}\text{|}\mathbf{u}_{j},\mathbf{z}_{j},\mathbf{\tau},\mathbf{\xi}\right) \\ \text{ }=\frac{\alpha_{i}-1}{c_{i}}-\frac{\beta_{i}-1}{1-c_{i}}+\frac{\sum_{k=1}^{q} \left(\bar{r}_{ik}-\bar{r}_{ik}^{(Z)}\right)}{c_{i}}-\frac{\sum_{k=1}^{q} \bar{f}_{ik}^{(Z)}}{1-c_{i}} \end{matrix}$$

Set Equation A.24 to 0 and solve for the estimate of $c{}_{i}$ which leads to a closed solution. The derivation is as follows:

$$\begin{matrix} \Rightarrow\frac{\alpha_{i}-1+\sum_{k=1}^{q} \left(\bar{r}_{ik}-\bar{r}_{ik}^{(Z)}\right)}{c_{i}}=\frac{\beta_{i}-1+\sum_{k=1}^{q} \bar{f}_{ik}^{(Z)}}{1-c_{i}} \\ \Rightarrow c_{i}=\frac{\alpha_{i}-1+\sum_{k=1}^{q} \left(\bar{r}_{ik}-\bar{r}_{ik}^{(Z)}\right)}{\alpha_{i}+\beta_{i}-2+\sum_{k=1}^{q} \left(\bar{f}_{ik}^{(Z)}+\bar{r}_{ik}-\bar{r}_{ik}^{(Z)}\right)} \\ \text{ }=\frac{\alpha_{i}-1+\sum_{k=1}^{q} \left(\bar{r}_{ik}-\bar{r}_{ik}^{(Z)}\right)}{\alpha_{i}+\beta_{i}-2+N} \end{matrix}$$

The second derivative is still useful for estimating SEs and is given below:

$$\begin{matrix} \lambda_{cc_{i}}=E[\frac{\partial^{2}\ln E\left[L\right]}{\partial^{2}c_{i}^{2}}|u_{ij}]+\frac{\partial^{2}\ln g(c_{i}|\alpha_{i},\beta_{i})}{\partial^{2}c_{i}^{2}} \\ \text{ }\approx E[\frac{\partial}{\partial c_{i}}\sum_{k=1}^{q} \left[\frac{\bar{r}_{ik}-\bar{r}_{ik}^{(Z)}}{c_{i}}-\frac{\bar{f}_{ik}^{(Z)}}{1-c_{i}}\right]|u_{ij}]-\frac{\alpha_{i}-1}{c_{i}^{2}}-\frac{\beta_{i}-1}{(1-c_{i})^{2}} \\ \text{ }=E[-\frac{\sum_{k=1}^{q} \bar{r}_{ik}-\bar{r}_{ik}^{(Z)}}{c_{i}^{2}}-\frac{\sum_{k=1}^{q} \bar{f}_{ik}^{(Z)}}{(1-c_{i})^{2}}|u_{ij}]-\frac{\alpha_{i}-1}{c_{i}^{2}}-\frac{\beta_{i}-1}{(1-c_{i})^{2}} \\ \text{ }=-\frac{\sum_{k=1}^{q} \bar{f}_{k}}{c_{i}(1-c_{i})}-\frac{\alpha_{i}-1}{c_{i}^{2}}-\frac{\beta_{i}-1}{(1-c_{i})^{2}}, \end{matrix}$$

since

$$\begin{matrix} E[u_{ij}|X_{k}]=p_{i}(X_{k})\text{ } \\ E[E(z_{ij}|u_{ij},X_{k},\mathbf{\xi})\text{ }|u_{ij}\text{]=}E(z_{ij}\text{)=}1-c_{i} \end{matrix}$$

and

$$\begin{matrix} E[\bar{f}_{k}|u_{ij}]=E[\sum_{j=1}^{N} P\left(X_{k}\text{|}\mathbf{u}_{j},\mathbf{z}_{j},\mathbf{\tau},\mathbf{\xi}\right)|u_{ij}] \\ \text{ }=\sum_{j=1}^{N} P\left(X_{k}\text{|}\mathbf{u}_{j},\mathbf{z}_{j},\mathbf{\tau},\mathbf{\xi}\right) \\ \text{ }=\bar{f}_{k}, \end{matrix}$$

$$\begin{matrix} \text{ }E[\bar{r}_{ik}|u_{ij}]=E[\sum_{j=1}^{N} u_{ij}P\left(X_{k}\text{|}\mathbf{u}_{j},\mathbf{z}_{j},\mathbf{\tau},\mathbf{\xi}\right)|u_{ij}] \\ \text{ }=p_{i}(X_{k})\sum_{j=1}^{N} P\left(X_{k}\text{|}\mathbf{u}_{j},\mathbf{z}_{j},\mathbf{\tau},\mathbf{\xi}\right)\text{ } \\ \text{ }=p_{i}(X_{k})\bar{f}_{k},\text{ } \end{matrix}$$

$$\begin{matrix} E[\bar{f}_{ik}^{(Z)}|u_{ij}]=E[\sum_{j=1}^{N} E(z_{ij}|u_{ij},X_{k},\mathbf{\xi})P\left(X_{k}\text{|}\mathbf{u}_{j},\mathbf{z}_{j},\mathbf{\tau},\mathbf{\xi}\right)|u_{ij}] \\ \text{ }=(1-c_{i})\sum_{j=1}^{N} P\left(X_{k}\text{|}\mathbf{u}_{j},\mathbf{z}_{j},\mathbf{\tau},\mathbf{\xi}\right)\text{ } \\ \text{ }=(1-c_{i})\bar{f}_{k}, \\ E[\bar{r}_{ik}^{(Z)}|u_{ij}]=E[\sum_{j=1}^{N} u_{ij}E(z_{ij}|u_{ij},X_{k},\mathbf{\xi})P\left(X_{k}\text{|}\mathbf{u}_{j},\mathbf{z}_{j},\mathbf{\tau},\mathbf{\xi}\right)|u_{ij}] \\ \text{ }=E[\sum_{j=1}^{N} (u_{ij}^{2}\times\frac{(1-c_{i})p_{i}^{*}(X_{k})}{p_{i}(X_{k})}+u_{ij}-u_{ij}^{2})P\left(X_{k}\text{|}\mathbf{u}_{j},\mathbf{z}_{j},\mathbf{\tau},\mathbf{\xi}\right)|u_{ij}] \\ \text{ }=\sum_{j=1}^{N} (p_{i}(X_{k})\times\frac{(1-c_{i})p_{i}^{*}(X_{k})}{p_{i}(X_{k})}+p_{i}(X_{k})-p_{i}(X_{k}))P\left(X_{k}\text{|}\mathbf{u}_{j},\mathbf{z}_{j},\mathbf{\tau},\mathbf{\xi}\right) \\ \text{ }=(1-c_{i})p_{i}^{*}(X_{k})\sum_{j=1}^{N} P\left(X_{k}\text{|}\mathbf{u}_{j},\mathbf{z}_{j},\mathbf{\tau},\mathbf{\xi}\right) \\ \text{ }=p_{i}^{*}(X_{k})\times(1-c_{i})\bar{f}_{k}. \end{matrix}$$

**Maximization Step-2 for *a* and *b* Parameters**

The second Maximization step is to execute the Fisher-scoring procedure to obtain estimates for $\ln a{}_{i}\text{ and }b{}_{i}$*.* The required first derivatives for $\ln a{}_{i}$ and $b{}_{i}$ are

The corresponding expectation of second derivatives are:

$$\begin{matrix} \lambda_{aa_{i}}=-D^{2}e^{2\ln a_{i}}\sum_{k=1}^{q} (X_{k}-b_{i})^{2}w_{ik}^{*}\times(1-c_{i})\bar{f}_{k}-\frac{1}{\sigma_{\ln a_{i}}^{2}}, \\ \lambda_{bb_{i}}=-D^{2}e^{2\ln a_{i}}\sum_{k=1}^{q} w_{ik}^{*}\times(1-c_{i})\bar{f}_{k}-\frac{1}{\sigma_{b_{i}}^{2}}, \\ \lambda_{ab_{i}}=D^{2}e^{2\ln a_{i}}\sum_{k=1}^{q} \left[(X_{k}-b_{i})w_{ik}^{*}\times(1-c_{i})\bar{f}_{k}\right], \end{matrix}$$

where

$$w_{ik}^{*}=p_{i}^{*}(X_{k})\times(1-p_{i}^{*}(X_{k})),$$

which lead to the Fisher-scoring algorithm for the BEMM:

$$\left[\begin{matrix} \ln a_{i}^{(t+1)} \\ b_{i}^{(t+1)} \end{matrix}\right]=\left[\begin{matrix} \ln a_{i}^{(t)} \\ b_{i}^{(t)} \end{matrix}\right]-\left[\begin{matrix} \lambda_{aa_{i}} & \lambda_{ab_{i}} \\ \lambda_{ab_{i}} & \lambda_{bb_{i}} \end{matrix}\right]^{-1}\left[\begin{matrix} \lambda_{a_{i}} \\ \lambda_{b_{i}} \end{matrix}\right].$$

In this case, estimation of the *c* parameters is separated from that of *a* and b, so the BEMM has a simplified 2-by-2 Hessian matrix (negative information matrix) in the iteration formulation.

# Appendix B

This Appendix provides the full details of the derivation from Equation 17, and all of the math notations is the same as in the article. Based on Equation 11, the joint probability can be obtained as:

$$L(\mathbf{U},\mathbf{V}|\mathbf{\xi})=\prod_{j=1}^{N} \int_{\theta_{j}} \left\{\prod_{i=1}^{n} P_{i}^{*}(\theta_{j})^{u_{ij}v_{ij}}\times\left[c_{i}\left(1-P_{i}^{*}(\theta_{j})\right)\right]^{u_{ij}(1-v_{ij})}\times[(1-c_{i})\left(1-P_{i}^{*}(\theta_{j})\right)]^{(1-u_{ij})\left(1-v_{ij}\right)}\right\}g(\theta_{j}|\mathbf{\tau})d\theta_{j}.$$

Furthermore, the joint distribution for the new augmented complete data $\left(\mathbf{U},\mathbf{V},\mathbf{\theta}\right)$ in which $\mathbf{\theta}=\left(\theta_{1},...\theta_{j},...\theta_{N}\right)$will be

$$P(\mathbf{u}_{j},\mathbf{v}_{j},\theta_{j}|\mathbf{\xi},\mathbf{\tau})=P(\mathbf{u}_{j},\mathbf{v}_{j}|\theta_{j},\mathbf{\xi})g(\theta_{j}|\mathbf{\tau}).$$

By integrating out the ability variable, the marginal distribution for a single examinee $j$ is

$$P(\mathbf{u}_{j},\mathbf{v}_{j}|\mathbf{\xi})=\int_{\theta_{j}} P(\mathbf{u}_{j},\mathbf{v}_{j}|\mathbf{\xi},\theta_{j})g(\theta_{j}|\mathbf{\tau})d\theta_{j}.$$

Thus, the likelihood function in Bayesian EMM (Equation 11) can be calculated from

$$\begin{matrix} L=\prod_{j=1}^{N} P(\mathbf{u}_{j},\mathbf{v}_{j}|\mathbf{\xi}) \\ \text{ }=\prod_{j=1}^{N} \int_{\theta_{j}} P(\mathbf{u}_{j},\mathbf{v}_{j}|\mathbf{\xi},\theta_{j})g(\theta_{j}|\mathbf{\tau})d\theta_{j} \\ \text{ }=\prod_{j=1}^{N} \int_{\theta_{j}} \left\{\prod_{i=1}^{n} p_{i}^{*}(\theta_{j})^{u_{ij}v_{ij}}\times\left[c_{i}\left(1-p_{i}^{*}(\theta_{j})\right)\right]^{u_{ij}(1-v_{ij})}\times[(1-c_{i})\left(1-p_{i}^{*}(\theta_{j})\right)]^{(1-u_{ij})\left(1-v_{ij}\right)}\right\}g(\theta_{j}|\mathbf{\tau})d\theta_{j}. \end{matrix}$$

Then, the first derivative of item parameters from log-likelihood function can be derived as

$$\begin{matrix} \text{Let }R_{h}\text{=}p_{h}^{*}(\theta_{j})^{u_{hj}v_{hj}}\times\left[c_{h}\left(1-p_{h}^{*}(\theta_{j})\right)\right]^{u_{hj}(1-v_{hj})}\times[(1-c_{h})\left(1-p_{h}^{*}(\theta_{j})\right)]^{(1-u_{hj})\left(1-v_{hj}\right)},\text{ } \\ \text{and }R_{i}\text{=}p_{i}^{*}(\theta_{j})^{u_{ij}v_{ij}}\times\left[c_{i}\left(1-p_{i}^{*}(\theta_{j})\right)\right]^{u_{ij}(1-v_{ij})}\times[(1-c_{i})\left(1-p_{i}^{*}(\theta_{j})\right)]^{(1-u_{ij})\left(1-v_{ij}\right)}\text{, so we have} \\ \frac{\partial\ln L}{\partial\psi_{i}}=\sum_{j=1}^{N} \int_{\theta_{j}} \frac{\frac{\partial}{\partial\psi_{i}}\left[R_{i}\times\prod_{h\neq i}^{n} R_{h}\right]}{R_{i}\times\prod_{h\neq i}^{n} R_{h}}P\left(\theta_{j}\text{|}\mathbf{u}_{j},\mathbf{v}_{j},\mathbf{\tau},\mathbf{\xi}\right)d\theta_{j} \\ \text{ =}\sum_{j=1}^{N} \int_{\theta_{j}} \frac{\left[\frac{\partial R_{i}}{\partial\psi_{i}}\right]\times\prod_{h\neq i}^{n} R_{h}+\left[R_{i}\times\frac{\partial}{\partial\psi_{i}}\prod_{h\neq i}^{n} R_{h}\right]}{R_{i}\times\prod_{h\neq i}^{n} R_{h}}P\left(\theta_{j}\text{|}\mathbf{u}_{j},\mathbf{v}_{j},\mathbf{\tau},\mathbf{\xi}\right)d\theta_{j}\text{ } \\ \text{ =}\sum_{j=1}^{N} \int_{\theta_{j}} \left[\frac{1}{R_{i}\times\prod_{h\neq i}^{n} R_{h}}\left(\left[\frac{\partial R_{i}}{\partial\psi_{i}}\right]\times\prod_{h\neq i}^{n} R_{h}+R_{i}\times0\right)\right]P\left(\theta_{j}\text{|}\mathbf{u}_{j},\mathbf{v}_{j},\mathbf{\tau},\mathbf{\xi}\right)d\theta_{j} \\ \text{ }=\sum_{j=1}^{N} \int_{\theta_{j}} \left[\frac{1}{R_{i}}\frac{\partial R_{i}}{\partial\psi_{i}}\right]P\left(\theta_{j}\text{|}\mathbf{u}_{j},\mathbf{v}_{j},\mathbf{\tau},\mathbf{\xi}\right)d\theta_{j} \\ \text{ }=\sum_{j=1}^{N} \int_{\theta_{j}} \left[\frac{1}{R_{i}}\frac{\partial p_{i}^{*}(\theta_{j})^{u_{ij}v_{ij}}\times\left[c_{i}\left(1-p_{i}^{*}(\theta_{j})\right)\right]^{u_{ij}(1-v_{ij})}\times[(1-c_{i})\left(1-p_{i}^{*}(\theta_{j})\right)]^{(1-u_{ij})\left(1-v_{ij}\right)}}{\partial\psi_{i}}\right]P\left(\theta_{j}\text{|}\mathbf{u}_{j},\mathbf{v}_{j},\mathbf{\tau},\mathbf{\xi}\right)d\theta_{j} \end{matrix}$$

$=\sum_{j=1}^{N} \int_{\theta_{j}} \left[\frac{1}{R_{i}}\left(\begin{matrix} \frac{\partial p_{i}^{*}(\theta_{j})}{\partial\psi_{i}}u_{ij}v_{ij}[p_{i}^{*}(\theta_{j})]^{u_{ij}v_{ij}-1}\times\left[c_{i}\left(1-p_{i}^{*}(\theta_{j})\right)\right]^{u_{ij}(1-v_{ij})}\times[(1-c_{i})\left(1-p_{i}^{*}(\theta_{j})\right)]^{(1-u_{ij})\left(1-v_{ij}\right)} \\ +p_{i}^{*}(\theta_{j})^{u_{ij}v_{ij}}\times\frac{\partial c_{i}\left(1-p_{i}^{*}(\theta_{j})\right)}{\partial\psi_{i}}u_{ij}(1-v_{ij})\left[c_{i}\left(1-p_{i}^{*}(\theta_{j})\right)\right]^{u_{ij}(1-v_{ij})-1}\times[(1-c_{i})\left(1-p_{i}^{*}(\theta_{j})\right)]^{(1-u_{ij})\left(1-v_{ij}\right)} \\ +p_{i}^{*}(\theta_{j})^{u_{ij}v_{ij}}\times\left[c_{i}\left(1-p_{i}^{*}(\theta_{j})\right)\right]^{u_{ij}(1-v_{ij})}\times\frac{\partial(1-c_{i})\left(1-p_{i}^{*}(\theta_{j})\right)}{\partial\psi_{i}}(1-u_{ij})\left(1-v_{ij}\right)[(1-c_{i})\left(1-p_{i}^{*}(\theta_{j})\right)]^{(1-u_{ij})\left(1-v_{ij}\right)-1} \end{matrix}\right)\right]P\left(\theta_{j}\text{|}\mathbf{u}_{j},\mathbf{v}_{j},\mathbf{\tau},\mathbf{\xi}\right)d\theta_{j}$

where

$$\begin{matrix} P\left(\theta_{j}\text{|}\mathbf{u}_{j},\mathbf{v}_{j},\mathbf{\tau},\mathbf{\xi}\right)=P\left(\theta_{j}\text{|}\mathbf{u}_{j},\mathbf{\tau},\mathbf{\xi}\right)=\frac{P(\mathbf{u}_{j}|\theta_{j},\mathbf{\xi})g(\theta_{j}|\mathbf{\tau})}{\int_{\theta_{j}} P(\mathbf{u}_{j}|\theta_{j},\mathbf{\xi})g(\theta_{j}|\mathbf{\tau})d\theta_{j}}, \\ P(\mathbf{u}_{j}|\theta_{j},\mathbf{\xi})=\prod_{i=1}^{n} p_{i}(\theta_{j})^{u_{ij}}\times(1-p_{i}(\theta_{j}))^{1-u_{ij}}, \end{matrix}$$

The conditional expectation of $v_{ij}$ is

$$E(v_{ij}|u_{ij},\theta_{j},\mathbf{\xi}_{i})=\frac{p_{i}^{*}(\theta_{j})}{p_{i}(\theta_{j})}\times u_{ij}+0\times(1-u_{ij}).$$

Plugging the conditional expectation of the indicator variable $\mathbf{V}$ and integrating over the latent ability variable just as in the original MMLE/EM. Finally, we obtained the Equation

$\frac{\partial\ln E\left[L\right]}{\partial\psi_{i}}\approx\sum_{j=1}^{N} \sum_{k=1}^{q} \left[\begin{matrix} \frac{u_{ij}E(v_{ij}|u_{ij},X_{k},\mathbf{\xi}_{i})}{P_{i}^{*}(X_{k})}\frac{\partial p_{i}^{*}(X_{k})}{\partial\psi_{i}} \\ -\frac{1-E(v_{ij}|u_{ij},X_{k},\mathbf{\xi}_{i})}{1-P_{i}^{*}(X_{k})}\frac{\partial p_{i}^{*}(X_{k})}{\partial\psi_{i}} \\ +\frac{u_{ij}(1-E(v_{ij}|u_{ij},X_{k},\mathbf{\xi}_{i}))}{c_{i}}\frac{\partial c_{i}}{\partial\psi_{i}} \\ -\frac{\left(1-u_{ij}\right)(1-E(v_{ij}|u_{ij},X_{k},\mathbf{\xi}_{i}))}{1-c_{i}}\frac{\partial c_{i}}{\partial\psi_{i}} \end{matrix}\right]P\left(X_{k}\text{|}\mathbf{u}_{j},\mathbf{v}_{j},\mathbf{\tau},\mathbf{\xi}\right)$,

with

$$\begin{matrix} P\left(X_{k}\text{|}\mathbf{u}_{j},\mathbf{v}_{j},\mathbf{\tau},\mathbf{\xi}\right)=P\left(X_{k}\text{|}\mathbf{u}_{j},\mathbf{\tau},\mathbf{\xi}\right)\text{=}\frac{P(\mathbf{u}_{j}|X_{k},\mathbf{\xi})\times A\left(X_{k}\right)}{\sum_{k=1}^{q} P(\mathbf{u}_{j}|X_{k},\mathbf{\xi})\times A\left(X_{k}\right)}, \\ P(\mathbf{u}_{j}|X_{k},\mathbf{\xi})=\prod_{i=1}^{n} P_{i}(X_{k})^{u_{ij}}\times(1-P_{i}(X_{k}))^{1-u_{ij}}, \end{matrix}$$

# Appendix C

Table C.1. RMSEs for item parameter estimate with 1000 examinees and 10 items

| Item | Generating | | | RMSEs for *a* | | | | RMSEs for *b* | | | | RMSEs for *c* | | | |
| --- | --- | --- | --- | --- | --- | --- | --- | --- | --- | --- | --- | --- | --- | --- | --- |
|  |  |  |  | Bayesian EMM | | BILOG-MG | | Bayesian EMM | | BILOG-MG | | Bayesian EMM | | BILOG-MG | |
|  | *a* | *b* | *c* | *Beta*(4,16) | *Beta*(1,4) | *Beta*(4,16) | *Beta*(1,4) | *Beta*(4,16) | *Beta*(1,4) | *Beta*(4,16) | *Beta*(1,4) | *Beta*(4,16) | *Beta*(1,4) | *Beta*(4,16) | *Beta*(1,4) |
| 1 | 1.731 | 1.012 | 0.280 | 0.501 | 0.495 | 0.436 | 0.425 | 0.104 | 0.106 | 0.093 | 0.094 | 0.047 | 0.048 | 0.041 | 0.041 |
| 2 | 0.989 | -0.858 | 0.134 | 0.163 | 0.155 | 0.182 | 0.195 | 0.115 | 0.115 | 0.138 | 0.160 | 0.039 | 0.043 | 0.055 | 0.069 |
| 3 | 1.140 | 0.081 | 0.157 | 0.155 | 0.169 | 0.160 | 0.175 | 0.086 | 0.123 | 0.081 | 0.109 | 0.033 | 0.052 | 0.034 | 0.048 |
| 4 | 0.481 | 1.364 | 0.218 | 0.162 | 0.183 | 0.233 | 0.277 | 0.242 | 0.272 | 0.218 | 0.224 | 0.042 | 0.054 | 0.057 | 0.069 |
| 5 | 0.760 | -0.394 | 0.226 | 0.087 | 0.093 | 0.096 | 0.129 | 0.139 | 0.178 | 0.119 | 0.180 | 0.051 | 0.074 | 0.042 | 0.077 |
| 6 | 1.800 | 0.065 | 0.186 | 0.326 | 0.358 | 0.310 | 0.333 | 0.098 | 0.121 | 0.090 | 0.107 | 0.037 | 0.051 | 0.033 | 0.044 |
| 7 | 2.394 | -1.062 | 0.160 | 0.381 | 0.421 | 0.470 | 0.519 | 0.058 | 0.081 | 0.070 | 0.105 | 0.021 | 0.055 | 0.019 | 0.058 |
| 8 | 0.826 | 0.084 | 0.225 | 0.099 | 0.111 | 0.114 | 0.143 | 0.153 | 0.193 | 0.133 | 0.175 | 0.053 | 0.075 | 0.047 | 0.072 |
| 9 | 1.555 | 0.901 | 0.233 | 0.358 | 0.366 | 0.338 | 0.340 | 0.091 | 0.096 | 0.085 | 0.088 | 0.032 | 0.036 | 0.028 | 0.031 |
| 10 | 0.974 | 0.991 | 0.140 | 0.214 | 0.221 | 0.227 | 0.227 | 0.109 | 0.127 | 0.107 | 0.118 | 0.030 | 0.042 | 0.030 | 0.036 |

Table C.2. RMSEs for item parameter estimate with 1500 examinees and 10 items

| Item | Generating | | | Bias for *a* | | | | Bias for *b* | | | | Bias for *c* | | | |
| --- | --- | --- | --- | --- | --- | --- | --- | --- | --- | --- | --- | --- | --- | --- | --- |
|  |  |  |  | Bayesian EMM | | BILOG-MG | | Bayesian EMM | | BILOG-MG | | Bayesian EMM | | BILOG-MG | |
|  | *a* | *b* | *c* | *Beta*(4,16) | *Beta*(1,4) | *Beta*(4,16) | *Beta*(1,4) | *Beta*(4,16) | *Beta*(1,4) | *Beta*(4,16) | *Beta*(1,4) | *Beta*(4,16) | *Beta*(1,4) | *Beta*(4,16) | *Beta*(1,4) |
| 1 | 1.158 | 1.704 | 0.194 | 0.235 | 0.247 | 0.228 | 0.238 | 0.123 | 0.124 | 0.124 | 0.124 | 0.023 | 0.026 | 0.022 | 0.024 |
| 2 | 1.139 | -0.529 | 0.184 | 0.092 | 0.095 | 0.095 | 0.120 | 0.067 | 0.089 | 0.071 | 0.127 | 0.026 | 0.040 | 0.032 | 0.066 |
| 3 | 0.940 | 0.403 | 0.241 | 0.135 | 0.150 | 0.136 | 0.160 | 0.141 | 0.163 | 0.133 | 0.164 | 0.053 | 0.063 | 0.050 | 0.064 |
| 4 | 1.500 | 1.087 | 0.120 | 0.188 | 0.199 | 0.184 | 0.194 | 0.052 | 0.052 | 0.053 | 0.053 | 0.015 | 0.018 | 0.015 | 0.017 |
| 5 | 0.508 | -0.969 | 0.199 | 0.055 | 0.055 | 0.070 | 0.149 | 0.128 | 0.138 | 0.180 | 0.495 | 0.015 | 0.023 | 0.041 | 0.148 |
| 6 | 1.446 | 0.964 | 0.189 | 0.255 | 0.267 | 0.253 | 0.264 | 0.064 | 0.067 | 0.063 | 0.065 | 0.024 | 0.026 | 0.023 | 0.025 |
| 7 | 1.558 | 0.131 | 0.307 | 0.303 | 0.308 | 0.287 | 0.293 | 0.174 | 0.173 | 0.158 | 0.156 | 0.072 | 0.073 | 0.066 | 0.067 |
| 8 | 2.255 | 0.267 | 0.174 | 0.370 | 0.399 | 0.365 | 0.387 | 0.065 | 0.075 | 0.063 | 0.073 | 0.029 | 0.035 | 0.028 | 0.034 |
| 9 | 1.368 | 0.051 | 0.216 | 0.184 | 0.204 | 0.182 | 0.203 | 0.094 | 0.111 | 0.091 | 0.112 | 0.043 | 0.053 | 0.042 | 0.054 |
| 10 | 1.622 | -0.558 | 0.199 | 0.166 | 0.181 | 0.172 | 0.211 | 0.078 | 0.098 | 0.083 | 0.129 | 0.033 | 0.049 | 0.035 | 0.070 |

Table C.3.

RMSEs for item parameter estimate with 2000 examinees and 10 items

| Item | Generating | | | RMSEs for *a* | | | | RMSEs for *b* | | | | RMSEs for *c* | | | |
| --- | --- | --- | --- | --- | --- | --- | --- | --- | --- | --- | --- | --- | --- | --- | --- |
|  |  |  |  | Bayesian EMM | | BILOG-MG | | Bayesian EMM | | BILOG-MG | | Bayesian EMM | | BILOG-MG | |
|  | *a* | *b* | *c* | *Beta*(4,16) | *Beta*(1,4) | *Beta*(4,16) | *Beta*(1,4) | *Beta*(4,16) | *Beta*(1,4) | *Beta*(4,16) | *Beta*(1,4) | *Beta*(4,16) | *Beta*(1,4) | *Beta*(4,16) | *Beta*(1,4) |
| 1 | 1.732 | 1.533 | 0.161 | 0.303 | 0.309 | 0.278 | 0.287 | 0.070 | 0.071 | 0.069 | 0.069 | 0.014 | 0.015 | 0.014 | 0.015 |
| 2 | 1.459 | 1.512 | 0.159 | 0.292 | 0.292 | 0.293 | 0.291 | 0.082 | 0.083 | 0.080 | 0.081 | 0.013 | 0.014 | 0.013 | 0.014 |
| 3 | 1.541 | 0.089 | 0.198 | 0.194 | 0.210 | 0.190 | 0.207 | 0.066 | 0.075 | 0.070 | 0.079 | 0.031 | 0.038 | 0.032 | 0.038 |
| 4 | 0.717 | 1.867 | 0.206 | 0.143 | 0.150 | 0.171 | 0.180 | 0.135 | 0.136 | 0.127 | 0.127 | 0.025 | 0.028 | 0.025 | 0.027 |
| 5 | 0.614 | -2.058 | 0.279 | 0.063 | 0.062 | 0.065 | 0.065 | 0.215 | 0.223 | 0.210 | 0.199 | 0.091 | 0.097 | 0.078 | 0.074 |
| 6 | 1.302 | 1.770 | 0.147 | 0.325 | 0.322 | 0.326 | 0.322 | 0.107 | 0.107 | 0.103 | 0.103 | 0.012 | 0.012 | 0.011 | 0.012 |
| 7 | 1.166 | -0.174 | 0.186 | 0.105 | 0.119 | 0.104 | 0.130 | 0.065 | 0.081 | 0.074 | 0.106 | 0.030 | 0.042 | 0.033 | 0.053 |
| 8 | 1.062 | 0.065 | 0.185 | 0.118 | 0.125 | 0.133 | 0.147 | 0.083 | 0.091 | 0.098 | 0.112 | 0.030 | 0.037 | 0.035 | 0.045 |
| 9 | 2.129 | 0.253 | 0.176 | 0.354 | 0.377 | 0.348 | 0.368 | 0.049 | 0.053 | 0.051 | 0.054 | 0.029 | 0.034 | 0.027 | 0.032 |
| 10 | 1.583 | -0.889 | 0.235 | 0.216 | 0.227 | 0.210 | 0.229 | 0.105 | 0.121 | 0.103 | 0.144 | 0.062 | 0.074 | 0.060 | 0.095 |

Table C.4.

RMSEs for item parameter estimate with 1000 examinees and 20 items

| Item | Generating | | | RMSEs for *a* | | | | RMSEs for *b* | | | | RMSEs for *c* | | | |
| --- | --- | --- | --- | --- | --- | --- | --- | --- | --- | --- | --- | --- | --- | --- | --- |
|  |  |  |  | Bayesian EMM | | BILOG-MG | | Bayesian EMM | | BILOG-MG | | Bayesian EMM | | BILOG-MG | |
|  | *a* | *b* | *c* | *Beta*(4,16) | *Beta*(1,4) | *Beta*(4,16) | *Beta*(1,4) | *Beta*(4,16) | *Beta*(1,4) | *Beta*(4,16) | *Beta*(1,4) | *Beta*(4,16) | *Beta*(1,4) | *Beta*(4,16) | *Beta*(1,4) |
| 1 | 1.547 | 0.540 | 0.292 | 0.354 | 0.376 | 0.315 | 0.319 | 0.100 | 0.101 | 0.090 | 0.088 | 0.046 | 0.047 | 0.042 | 0.042 |
| 2 | 0.605 | 0.705 | 0.182 | 0.132 | 0.132 | 0.157 | 0.173 | 0.133 | 0.175 | 0.147 | 0.183 | 0.038 | 0.053 | 0.047 | 0.061 |
| 3 | 0.692 | 0.592 | 0.143 | 0.160 | 0.149 | 0.176 | 0.173 | 0.097 | 0.118 | 0.117 | 0.123 | 0.037 | 0.043 | 0.045 | 0.047 |
| 4 | 0.958 | -0.160 | 0.216 | 0.134 | 0.155 | 0.148 | 0.181 | 0.118 | 0.156 | 0.128 | 0.173 | 0.045 | 0.067 | 0.045 | 0.069 |
| 5 | 0.581 | 0.393 | 0.189 | 0.138 | 0.145 | 0.174 | 0.206 | 0.144 | 0.189 | 0.185 | 0.241 | 0.044 | 0.062 | 0.061 | 0.082 |
| 6 | 1.477 | 0.909 | 0.151 | 0.237 | 0.235 | 0.244 | 0.241 | 0.080 | 0.088 | 0.072 | 0.077 | 0.024 | 0.028 | 0.023 | 0.026 |
| 7 | 1.076 | 1.634 | 0.232 | 0.271 | 0.285 | 0.267 | 0.278 | 0.172 | 0.173 | 0.158 | 0.159 | 0.034 | 0.038 | 0.030 | 0.033 |
| 8 | 1.548 | 2.107 | 0.106 | 0.372 | 0.383 | 0.317 | 0.373 | 0.182 | 0.181 | 0.171 | 0.174 | 0.012 | 0.014 | 0.011 | 0.013 |
| 9 | 0.911 | -0.791 | 0.189 | 0.113 | 0.112 | 0.127 | 0.167 | 0.108 | 0.123 | 0.141 | 0.212 | 0.030 | 0.055 | 0.038 | 0.083 |
| 10 | 0.480 | 0.064 | 0.123 | 0.132 | 0.128 | 0.165 | 0.199 | 0.275 | 0.270 | 0.382 | 0.449 | 0.077 | 0.076 | 0.109 | 0.131 |
| 11 | 0.480 | -0.547 | 0.195 | 0.098 | 0.096 | 0.117 | 0.186 | 0.130 | 0.143 | 0.207 | 0.438 | 0.021 | 0.038 | 0.042 | 0.119 |
| 12 | 0.509 | 1.533 | 0.126 | 0.220 | 0.204 | 0.241 | 0.236 | 0.174 | 0.193 | 0.174 | 0.180 | 0.049 | 0.045 | 0.058 | 0.056 |
| 13 | 2.081 | 0.886 | 0.122 | 0.361 | 0.358 | 0.311 | 0.316 | 0.059 | 0.065 | 0.051 | 0.055 | 0.020 | 0.023 | 0.019 | 0.021 |
| 14 | 2.234 | 1.458 | 0.198 | 0.432 | 0.438 | 0.418 | 0.419 | 0.092 | 0.092 | 0.084 | 0.085 | 0.017 | 0.017 | 0.016 | 0.017 |
| 15 | 0.651 | 0.229 | 0.294 | 0.081 | 0.093 | 0.114 | 0.190 | 0.294 | 0.335 | 0.256 | 0.323 | 0.092 | 0.107 | 0.075 | 0.096 |
| 16 | 0.872 | -0.539 | 0.075 | 0.180 | 0.161 | 0.199 | 0.196 | 0.221 | 0.189 | 0.257 | 0.240 | 0.091 | 0.078 | 0.107 | 0.099 |
| 17 | 0.532 | 0.152 | 0.128 | 0.140 | 0.127 | 0.170 | 0.186 | 0.210 | 0.212 | 0.281 | 0.318 | 0.062 | 0.064 | 0.086 | 0.098 |
| 18 | 1.319 | -0.277 | 0.310 | 0.186 | 0.207 | 0.174 | 0.205 | 0.163 | 0.183 | 0.141 | 0.162 | 0.095 | 0.103 | 0.080 | 0.084 |
| 19 | 1.141 | 0.558 | 0.227 | 0.192 | 0.211 | 0.197 | 0.221 | 0.118 | 0.136 | 0.108 | 0.123 | 0.043 | 0.054 | 0.040 | 0.048 |
| 20 | 0.666 | 1.510 | 0.233 | 0.257 | 0.276 | 0.276 | 0.299 | 0.210 | 0.224 | 0.189 | 0.195 | 0.040 | 0.048 | 0.038 | 0.044 |

Table C.5

RMSEs for item parameter estimate with 1500 examinees and 20 items

| Item | Generating | | | RMSEs for *a* | | | | RMSEs for *b* | | | | RMSEs for *c* | | | |
| --- | --- | --- | --- | --- | --- | --- | --- | --- | --- | --- | --- | --- | --- | --- | --- |
|  |  |  |  | Bayesian EMM | | BILOG-MG | | Bayesian EMM | | BILOG-MG | | Bayesian EMM | | BILOG-MG | |
|  | *a* | *b* | *c* | *Beta*(4,16) | *Beta*(1,4) | *Beta*(4,16) | *Beta*(1,4) | *Beta*(4,16) | *Beta*(1,4) | *Beta*(4,16) | *Beta*(1,4) | *Beta*(4,16) | *Beta*(1,4) | *Beta*(4,16) | *Beta*(1,4) |
| 1 | 0.783 | -0.473 | 0.156 | 0.075 | 0.075 | 0.092 | 0.106 | 0.097 | 0.120 | 0.125 | 0.177 | 0.039 | 0.047 | 0.056 | 0.077 |
| 2 | 0.722 | 1.013 | 0.189 | 0.125 | 0.138 | 0.129 | 0.141 | 0.147 | 0.175 | 0.134 | 0.156 | 0.039 | 0.051 | 0.037 | 0.047 |
| 3 | 1.597 | -0.543 | 0.184 | 0.178 | 0.197 | 0.184 | 0.209 | 0.085 | 0.107 | 0.081 | 0.107 | 0.042 | 0.057 | 0.042 | 0.060 |
| 4 | 1.256 | 1.534 | 0.307 | 0.330 | 0.326 | 0.292 | 0.286 | 0.122 | 0.119 | 0.118 | 0.116 | 0.031 | 0.028 | 0.024 | 0.022 |
| 5 | 2.485 | 0.666 | 0.150 | 0.363 | 0.377 | 0.355 | 0.367 | 0.069 | 0.072 | 0.063 | 0.065 | 0.014 | 0.015 | 0.013 | 0.014 |
| 6 | 1.629 | -0.230 | 0.273 | 0.175 | 0.191 | 0.167 | 0.184 | 0.144 | 0.152 | 0.130 | 0.136 | 0.066 | 0.074 | 0.061 | 0.068 |
| 7 | 0.864 | -0.326 | 0.148 | 0.104 | 0.105 | 0.118 | 0.126 | 0.102 | 0.129 | 0.115 | 0.148 | 0.043 | 0.052 | 0.053 | 0.065 |
| 8 | 2.177 | -0.685 | 0.248 | 0.317 | 0.331 | 0.301 | 0.318 | 0.124 | 0.136 | 0.112 | 0.127 | 0.068 | 0.081 | 0.061 | 0.078 |
| 9 | 2.021 | -0.132 | 0.188 | 0.309 | 0.323 | 0.312 | 0.324 | 0.080 | 0.091 | 0.072 | 0.081 | 0.036 | 0.044 | 0.034 | 0.041 |
| 10 | 1.963 | 0.185 | 0.206 | 0.251 | 0.262 | 0.250 | 0.260 | 0.089 | 0.094 | 0.081 | 0.084 | 0.031 | 0.034 | 0.030 | 0.032 |
| 11 | 0.572 | 1.003 | 0.162 | 0.135 | 0.136 | 0.153 | 0.158 | 0.131 | 0.155 | 0.133 | 0.153 | 0.038 | 0.044 | 0.045 | 0.050 |
| 12 | 1.497 | 1.418 | 0.196 | 0.310 | 0.314 | 0.307 | 0.310 | 0.087 | 0.087 | 0.085 | 0.085 | 0.017 | 0.018 | 0.016 | 0.017 |
| 13 | 1.256 | 2.217 | 0.182 | 0.258 | 0.271 | 0.248 | 0.258 | 0.161 | 0.163 | 0.173 | 0.174 | 0.017 | 0.019 | 0.017 | 0.018 |
| 14 | 0.813 | -0.105 | 0.208 | 0.096 | 0.110 | 0.111 | 0.133 | 0.133 | 0.167 | 0.124 | 0.166 | 0.043 | 0.059 | 0.045 | 0.065 |
| 15 | 0.759 | 2.227 | 0.166 | 0.221 | 0.225 | 0.222 | 0.223 | 0.198 | 0.197 | 0.196 | 0.196 | 0.022 | 0.026 | 0.022 | 0.024 |
| 16 | 0.972 | 2.231 | 0.240 | 0.221 | 0.227 | 0.209 | 0.211 | 0.213 | 0.213 | 0.224 | 0.223 | 0.020 | 0.021 | 0.018 | 0.018 |
| 17 | 1.190 | -0.118 | 0.214 | 0.119 | 0.131 | 0.117 | 0.129 | 0.122 | 0.144 | 0.107 | 0.127 | 0.045 | 0.058 | 0.040 | 0.052 |
| 18 | 0.986 | 0.541 | 0.105 | 0.119 | 0.131 | 0.123 | 0.127 | 0.073 | 0.107 | 0.064 | 0.088 | 0.026 | 0.037 | 0.027 | 0.032 |
| 19 | 1.658 | -0.379 | 0.174 | 0.171 | 0.189 | 0.175 | 0.197 | 0.081 | 0.102 | 0.072 | 0.094 | 0.035 | 0.049 | 0.033 | 0.047 |
| 20 | 0.572 | -1.208 | 0.127 | 0.070 | 0.068 | 0.079 | 0.114 | 0.165 | 0.152 | 0.221 | 0.380 | 0.060 | 0.052 | 0.086 | 0.141 |

Table C.6.

RMSE for item parameter estimate with 2000 examinees and 20 items

| Item | Generating | | | RMSEs for *a* | | | | RMSEs for *b* | | | | RMSEs for *c* | | | |
| --- | --- | --- | --- | --- | --- | --- | --- | --- | --- | --- | --- | --- | --- | --- | --- |
|  |  |  |  | Bayesian EMM | | BILOG-MG | | Bayesian EMM | | BILOG-MG | | Bayesian EMM | | BILOG-MG | |
|  | *a* | *b* | *c* | *Beta*(4,16) | *Beta*(1,4) | *Beta*(4,16) | *Beta*(1,4) | *Beta*(4,16) | *Beta*(1,4) | *Beta*(4,16) | *Beta*(1,4) | *Beta*(4,16) | *Beta*(1,4) | *Beta*(4,16) | *Beta*(1,4) |
| 1 | 1.050 | 1.351 | 0.321 | 0.224 | 0.224 | 0.189 | 0.191 | 0.093 | 0.094 | 0.101 | 0.104 | 0.035 | 0.034 | 0.025 | 0.023 |
| 2 | 2.437 | 0.413 | 0.182 | 0.267 | 0.274 | 0.265 | 0.270 | 0.035 | 0.035 | 0.040 | 0.040 | 0.016 | 0.017 | 0.015 | 0.016 |
| 3 | 2.213 | 1.507 | 0.153 | 0.419 | 0.428 | 0.416 | 0.425 | 0.076 | 0.076 | 0.081 | 0.081 | 0.011 | 0.011 | 0.010 | 0.011 |
| 4 | 1.157 | 1.291 | 0.192 | 0.177 | 0.186 | 0.172 | 0.179 | 0.077 | 0.077 | 0.080 | 0.080 | 0.024 | 0.027 | 0.022 | 0.024 |
| 5 | 1.488 | -0.960 | 0.172 | 0.135 | 0.136 | 0.139 | 0.149 | 0.045 | 0.056 | 0.049 | 0.077 | 0.030 | 0.046 | 0.032 | 0.063 |
| 6 | 1.152 | 0.796 | 0.166 | 0.148 | 0.151 | 0.150 | 0.151 | 0.061 | 0.061 | 0.067 | 0.066 | 0.019 | 0.021 | 0.019 | 0.020 |
| 7 | 0.581 | -0.678 | 0.176 | 0.054 | 0.054 | 0.072 | 0.101 | 0.101 | 0.113 | 0.175 | 0.268 | 0.026 | 0.034 | 0.054 | 0.089 |
| 8 | 1.917 | 0.341 | 0.178 | 0.199 | 0.205 | 0.196 | 0.201 | 0.042 | 0.042 | 0.046 | 0.046 | 0.019 | 0.020 | 0.018 | 0.019 |
| 9 | 0.861 | 0.894 | 0.223 | 0.120 | 0.129 | 0.118 | 0.126 | 0.083 | 0.094 | 0.080 | 0.090 | 0.034 | 0.040 | 0.031 | 0.035 |
| 10 | 1.160 | 0.389 | 0.239 | 0.134 | 0.139 | 0.128 | 0.132 | 0.073 | 0.077 | 0.068 | 0.071 | 0.029 | 0.032 | 0.026 | 0.028 |
| 11 | 1.094 | 0.242 | 0.278 | 0.128 | 0.132 | 0.118 | 0.124 | 0.100 | 0.106 | 0.094 | 0.103 | 0.043 | 0.045 | 0.038 | 0.039 |
| 12 | 0.332 | 0.089 | 0.175 | 0.050 | 0.048 | 0.098 | 0.145 | 0.154 | 0.151 | 0.419 | 0.642 | 0.025 | 0.026 | 0.088 | 0.138 |
| 13 | 0.557 | 0.308 | 0.151 | 0.064 | 0.060 | 0.084 | 0.089 | 0.119 | 0.118 | 0.176 | 0.194 | 0.035 | 0.035 | 0.054 | 0.061 |
| 14 | 2.248 | -0.622 | 0.279 | 0.372 | 0.377 | 0.344 | 0.343 | 0.122 | 0.130 | 0.110 | 0.117 | 0.077 | 0.084 | 0.068 | 0.075 |
| 15 | 1.931 | 0.853 | 0.242 | 0.248 | 0.247 | 0.245 | 0.244 | 0.049 | 0.050 | 0.053 | 0.054 | 0.015 | 0.015 | 0.014 | 0.014 |
| 16 | 0.551 | 0.661 | 0.138 | 0.067 | 0.059 | 0.085 | 0.082 | 0.135 | 0.136 | 0.174 | 0.182 | 0.034 | 0.036 | 0.047 | 0.051 |
| 17 | 0.860 | -0.283 | 0.137 | 0.066 | 0.066 | 0.071 | 0.074 | 0.074 | 0.082 | 0.088 | 0.098 | 0.028 | 0.037 | 0.032 | 0.044 |
| 18 | 1.072 | -1.032 | 0.110 | 0.098 | 0.091 | 0.102 | 0.098 | 0.103 | 0.091 | 0.115 | 0.109 | 0.048 | 0.040 | 0.057 | 0.055 |
| 19 | 0.481 | 1.099 | 0.214 | 0.055 | 0.061 | 0.099 | 0.120 | 0.184 | 0.212 | 0.216 | 0.250 | 0.041 | 0.052 | 0.049 | 0.062 |
| 20 | 1.100 | -1.004 | 0.126 | 0.120 | 0.116 | 0.127 | 0.130 | 0.097 | 0.088 | 0.111 | 0.116 | 0.040 | 0.037 | 0.051 | 0.060 |

Figure C.1.

Item parameter recovery for 1000 examinees with 10 items (RMSE)

Figure C.2.

Item parameter recovery for 1000 examinees with 20 items (RMSE)

Figure C.3.

Item parameter recovery for 1500 examinees with 10 items (RMSE)

Figure C.4.

Item parameter recovery for 1500 examinees with 20 items (RMSE)

Figure C.5.

Item parameter recovery for 2000 examinees with 10 items (RMSE)

Figure C.6.

Item parameter recovery for 2000 examinees with 20 items (RMSE)

| Table C.7 Bias for item parameter estimate with 1000 examinees and 10 items | | | | | | | | | | | | | | | |
| --- | --- | --- | --- | --- | --- | --- | --- | --- | --- | --- | --- | --- | --- | --- | --- |
| Item | Generating | | | Bias for *a* | | | | Bias for *b* | | | | Bias for *c* | | | |
|  |  |  |  | Bayesian EMM | | BILOG-MG | | Bayesian EMM | | BILOG-MG | | Bayesian EMM | | BILOG-MG | |
|  | *a* | *b* | *c* | *Beta*(4,16) | *Beta*(1,4) | *Beta*(4,16) | *Beta*(1,4) | *Beta*(4,16) | *Beta*(1,4) | *Beta*(4,16) | *Beta*(1,4) | *Beta*(4,16) | *Beta*(1,4) | *Beta*(4,16) | *Beta*(1,4) |
| 1 | 1.731 | 1.012 | 0.280 | -0.282 | -0.243 | -0.284 | -0.269 | -0.045 | -0.041 | -0.030 | -0.025 | -0.028 | -0.025 | -0.023 | -0.020 |
| 2 | 0.989 | -0.858 | 0.134 | 0.089 | 0.067 | 0.113 | 0.102 | 0.072 | 0.031 | 0.101 | 0.075 | 0.031 | 0.005 | 0.047 | 0.030 |
| 3 | 1.140 | 0.081 | 0.157 | 0.019 | -0.019 | 0.040 | 0.014 | -0.030 | -0.067 | -0.012 | -0.039 | -0.001 | -0.021 | 0.008 | -0.006 |
| 4 | 0.481 | 1.364 | 0.218 | 0.097 | 0.104 | 0.179 | 0.220 | -0.137 | -0.149 | -0.042 | -0.017 | 0.000 | -0.002 | 0.034 | 0.046 |
| 5 | 0.760 | -0.394 | 0.226 | -0.006 | -0.015 | 0.017 | 0.039 | -0.093 | -0.125 | -0.039 | -0.004 | -0.043 | -0.057 | -0.020 | -0.009 |
| 6 | 1.800 | 0.065 | 0.186 | -0.170 | -0.201 | -0.161 | -0.187 | -0.070 | -0.088 | -0.060 | -0.073 | -0.021 | -0.032 | -0.015 | -0.023 |
| 7 | 2.394 | -1.062 | 0.160 | -0.216 | -0.308 | -0.363 | -0.421 | -0.028 | -0.064 | -0.037 | -0.086 | -0.009 | -0.045 | 0.000 | -0.049 |
| 8 | 0.826 | 0.084 | 0.225 | 0.008 | 0.001 | 0.041 | 0.056 | -0.114 | -0.136 | -0.067 | -0.061 | -0.036 | -0.046 | -0.017 | -0.016 |
| 9 | 1.555 | 0.901 | 0.233 | -0.096 | -0.080 | -0.072 | -0.059 | -0.033 | -0.034 | -0.021 | -0.020 | -0.018 | -0.017 | -0.013 | -0.011 |
| 10 | 0.974 | 0.991 | 0.140 | 0.077 | 0.034 | 0.101 | 0.066 | -0.017 | -0.041 | -0.002 | -0.020 | 0.005 | -0.009 | 0.011 | 0.001 |

| Table C.8 Bias for item parameter estimate with 1500 examinees and 10 items | | | | | | | | | | | | | | | |
| --- | --- | --- | --- | --- | --- | --- | --- | --- | --- | --- | --- | --- | --- | --- | --- |
| Item | Generating | | | Bias for *a* | | | | Bias for *b* | | | | Bias for *c* | | | |
|  |  |  |  | Bayesian EMM | | BILOG-MG | | Bayesian EMM | | BILOG-MG | | Bayesian EMM | | BILOG-MG | |
|  | *a* | *b* | *c* | *Beta*(4,16) | *Beta*(1,4) | *Beta*(4,16) | *Beta*(1,4) | *Beta*(4,16) | *Beta*(1,4) | *Beta*(4,16) | *Beta*(1,4) | *Beta*(4,16) | *Beta*(1,4) | *Beta*(4,16) | *Beta*(1,4) |
| 1 | 1.158 | 1.704 | 0.194 | -0.049 | -0.057 | -0.037 | -0.040 | -0.019 | -0.020 | -0.009 | -0.011 | -0.009 | -0.011 | -0.007 | -0.008 |
| 2 | 1.139 | -0.529 | 0.184 | -0.002 | -0.016 | 0.008 | 0.001 | -0.032 | -0.053 | -0.022 | -0.042 | -0.006 | -0.018 | 0.001 | -0.012 |
| 3 | 0.940 | 0.403 | 0.241 | -0.036 | -0.032 | -0.008 | 0.010 | -0.081 | -0.085 | -0.052 | -0.047 | -0.029 | -0.031 | -0.018 | -0.016 |
| 4 | 1.500 | 1.087 | 0.120 | 0.011 | -0.030 | 0.013 | -0.028 | 0.006 | 0.001 | 0.011 | 0.007 | 0.000 | -0.004 | 0.002 | -0.003 |
| 5 | 0.508 | -0.969 | 0.199 | 0.019 | 0.017 | 0.034 | 0.095 | -0.004 | -0.020 | 0.076 | 0.358 | -0.003 | -0.009 | 0.023 | 0.107 |
| 6 | 1.446 | 0.964 | 0.189 | 0.039 | 0.036 | 0.039 | 0.037 | -0.017 | -0.020 | -0.012 | -0.014 | -0.003 | -0.005 | -0.002 | -0.003 |
| 7 | 1.558 | 0.131 | 0.307 | -0.181 | -0.140 | -0.143 | -0.086 | -0.130 | -0.114 | -0.109 | -0.085 | -0.051 | -0.043 | -0.041 | -0.029 |
| 8 | 2.255 | 0.267 | 0.174 | -0.178 | -0.207 | -0.186 | -0.219 | -0.037 | -0.045 | -0.035 | -0.042 | -0.009 | -0.014 | -0.007 | -0.012 |
| 9 | 1.368 | 0.051 | 0.216 | -0.044 | -0.049 | -0.036 | -0.037 | -0.043 | -0.051 | -0.036 | -0.042 | -0.019 | -0.024 | -0.016 | -0.019 |
| 10 | 1.622 | -0.558 | 0.199 | -0.058 | -0.081 | -0.052 | -0.088 | -0.045 | -0.063 | -0.044 | -0.081 | -0.021 | -0.033 | -0.019 | -0.046 |

| Table C.9 Bias for item parameter estimate with 2000 examinees and 10 items | | | | | | | | | | | | | | | |
| --- | --- | --- | --- | --- | --- | --- | --- | --- | --- | --- | --- | --- | --- | --- | --- |
| Item | Generating | | | Bias for *a* | | | | Bias for *b* | | | | Bias for *c* | | | |
|  |  |  |  | Bayesian EMM | | BILOG-MG | | Bayesian EMM | | BILOG-MG | | Bayesian EMM | | BILOG-MG | |
|  | *a* | *b* | *c* | *Beta*(4,16) | *Beta*(1,4) | *Beta*(4,16) | *Beta*(1,4) | *Beta*(4,16) | *Beta*(1,4) | *Beta*(4,16) | *Beta*(1,4) | *Beta*(4,16) | *Beta*(1,4) | *Beta*(4,16) | *Beta*(1,4) |
| 1 | 1.732 | 1.533 | 0.161 | -0.013 | -0.024 | -0.028 | -0.038 | -0.020 | -0.021 | -0.011 | -0.012 | -0.006 | -0.007 | -0.005 | -0.006 |
| 2 | 1.459 | 1.512 | 0.159 | 0.118 | 0.107 | 0.126 | 0.115 | -0.027 | -0.028 | -0.019 | -0.020 | -0.002 | -0.003 | -0.001 | -0.002 |
| 3 | 1.541 | 0.089 | 0.198 | -0.080 | -0.087 | -0.051 | -0.052 | 0.003 | -0.002 | 0.017 | 0.014 | -0.009 | -0.012 | -0.001 | -0.003 |
| 4 | 0.717 | 1.867 | 0.206 | 0.044 | 0.043 | 0.102 | 0.107 | -0.064 | -0.065 | -0.048 | -0.049 | -0.004 | -0.005 | 0.007 | 0.008 |
| 5 | 0.614 | -2.058 | 0.279 | 0.005 | 0.003 | 0.005 | 0.006 | -0.138 | -0.150 | -0.123 | -0.102 | -0.091 | -0.097 | -0.078 | -0.066 |
| 6 | 1.302 | 1.770 | 0.147 | 0.167 | 0.152 | 0.168 | 0.152 | -0.061 | -0.061 | -0.051 | -0.051 | -0.001 | -0.002 | 0.000 | -0.001 |
| 7 | 1.166 | -0.174 | 0.186 | -0.062 | -0.078 | -0.048 | -0.064 | -0.009 | -0.028 | 0.004 | -0.019 | -0.015 | -0.025 | -0.008 | -0.020 |
| 8 | 1.062 | 0.065 | 0.185 | 0.019 | 0.012 | 0.043 | 0.041 | 0.030 | 0.020 | 0.050 | 0.043 | 0.002 | -0.003 | 0.011 | 0.007 |
| 9 | 2.129 | 0.253 | 0.176 | -0.172 | -0.197 | -0.153 | -0.175 | 0.008 | 0.001 | 0.015 | 0.008 | -0.015 | -0.020 | -0.012 | -0.016 |
| 10 | 1.583 | -0.889 | 0.235 | -0.149 | -0.169 | -0.136 | -0.170 | -0.095 | -0.110 | -0.091 | -0.126 | -0.061 | -0.072 | -0.058 | -0.085 |

| Table C.10 Bias for item parameter estimate with 1000 examinees and 20 items | | | | | | | | | | | | | | | |
| --- | --- | --- | --- | --- | --- | --- | --- | --- | --- | --- | --- | --- | --- | --- | --- |
| Item | Generating | | | Bias for *a* | | | | Bias for *b* | | | | Bias for *c* | | | |
|  |  |  |  | Bayesian EMM | | BILOG-MG | | Bayesian EMM | | BILOG-MG | | Bayesian EMM | | BILOG-MG | |
|  | *a* | *b* | *c* | *Beta*(4,16) | *Beta*(1,4) | *Beta*(4,16) | *Beta*(1,4) | *Beta*(4,16) | *Beta*(1,4) | *Beta*(4,16) | *Beta*(1,4) | *Beta*(4,16) | *Beta*(1,4) | *Beta*(4,16) | *Beta*(1,4) |
| 1 | 1.547 | 0.540 | 0.292 | 0.051 | 0.092 | 0.051 | 0.089 | -0.044 | -0.033 | -0.026 | -0.012 | -0.019 | -0.012 | -0.015 | -0.008 |
| 2 | 0.605 | 0.705 | 0.182 | 0.089 | 0.074 | 0.120 | 0.128 | -0.017 | -0.055 | 0.045 | 0.041 | 0.009 | -0.005 | 0.028 | 0.026 |
| 3 | 0.692 | 0.592 | 0.143 | 0.113 | 0.081 | 0.135 | 0.117 | 0.036 | -0.015 | 0.077 | 0.045 | 0.023 | 0.003 | 0.036 | 0.023 |
| 4 | 0.958 | -0.160 | 0.216 | 0.044 | 0.043 | 0.068 | 0.086 | 0.002 | -0.012 | 0.042 | 0.051 | -0.012 | -0.019 | 0.004 | 0.007 |
| 5 | 0.581 | 0.393 | 0.189 | 0.089 | 0.082 | 0.127 | 0.148 | 0.016 | -0.018 | 0.096 | 0.110 | 0.011 | -0.001 | 0.035 | 0.040 |
| 6 | 1.477 | 0.909 | 0.151 | 0.131 | 0.101 | 0.144 | 0.121 | -0.045 | -0.053 | -0.028 | -0.035 | -0.002 | -0.007 | 0.001 | -0.003 |
| 7 | 1.076 | 1.634 | 0.232 | 0.040 | 0.039 | 0.055 | 0.062 | -0.132 | -0.133 | -0.109 | -0.111 | -0.018 | -0.019 | -0.014 | -0.014 |
| 8 | 1.548 | 2.107 | 0.106 | 0.018 | -0.028 | -0.008 | -0.028 | -0.121 | -0.116 | -0.098 | -0.099 | -0.002 | -0.005 | -0.001 | -0.004 |
| 9 | 0.911 | -0.791 | 0.189 | 0.061 | 0.050 | 0.079 | 0.098 | 0.058 | 0.027 | 0.098 | 0.106 | -0.006 | -0.024 | 0.014 | 0.014 |
| 10 | 0.480 | 0.064 | 0.123 | 0.107 | 0.096 | 0.142 | 0.165 | 0.227 | 0.178 | 0.329 | 0.351 | 0.068 | 0.051 | 0.098 | 0.104 |
| 11 | 0.480 | -0.547 | 0.195 | 0.071 | 0.065 | 0.090 | 0.144 | 0.055 | 0.015 | 0.153 | 0.331 | -0.002 | -0.016 | 0.029 | 0.081 |
| 12 | 0.509 | 1.533 | 0.126 | 0.182 | 0.148 | 0.207 | 0.192 | -0.042 | -0.077 | 0.002 | -0.017 | 0.042 | 0.026 | 0.053 | 0.045 |
| 13 | 2.081 | 0.886 | 0.122 | 0.093 | 0.042 | 0.093 | 0.054 | -0.036 | -0.044 | -0.020 | -0.027 | -0.001 | -0.006 | 0.001 | -0.003 |
| 14 | 2.234 | 1.458 | 0.198 | -0.269 | -0.276 | -0.336 | -0.335 | -0.057 | -0.057 | -0.040 | -0.042 | -0.005 | -0.006 | -0.004 | -0.005 |
| 15 | 0.651 | 0.229 | 0.294 | -0.020 | -0.017 | 0.036 | 0.107 | -0.246 | -0.254 | -0.107 | -0.001 | -0.082 | -0.085 | -0.039 | -0.006 |
| 16 | 0.872 | -0.539 | 0.075 | 0.143 | 0.112 | 0.161 | 0.140 | 0.203 | 0.146 | 0.237 | 0.190 | 0.082 | 0.050 | 0.098 | 0.070 |
| 17 | 0.532 | 0.152 | 0.128 | 0.122 | 0.104 | 0.152 | 0.157 | 0.148 | 0.089 | 0.221 | 0.207 | 0.049 | 0.027 | 0.072 | 0.066 |
| 18 | 1.319 | -0.277 | 0.310 | -0.105 | -0.080 | -0.068 | 0.002 | -0.119 | -0.106 | -0.076 | -0.024 | -0.084 | -0.077 | -0.063 | -0.039 |
| 19 | 1.141 | 0.558 | 0.227 | 0.062 | 0.063 | 0.081 | 0.092 | -0.048 | -0.054 | -0.024 | -0.023 | -0.017 | -0.019 | -0.010 | -0.009 |
| 20 | 0.666 | 1.510 | 0.233 | 0.133 | 0.137 | 0.173 | 0.195 | -0.123 | -0.130 | -0.078 | -0.074 | -0.003 | -0.005 | 0.009 | 0.013 |

| Table C.11 Bias for item parameter estimate with 1500 examinees and 20 items | | | | | | | | | | | | | | | |
| --- | --- | --- | --- | --- | --- | --- | --- | --- | --- | --- | --- | --- | --- | --- | --- |
| Item | Generating | | | Bias for *a* | | | | Bias for *b* | | | | Bias for *c* | | | |
|  |  |  |  | Bayesian EMM | | BILOG-MG | | Bayesian EMM | | BILOG-MG | | Bayesian EMM | | BILOG-MG | |
|  | *a* | *b* | *c* | *Beta*(4,16) | *Beta*(1,4) | *Beta*(4,16) | *Beta*(1,4) | *Beta*(4,16) | *Beta*(1,4) | *Beta*(4,16) | *Beta*(1,4) | *Beta*(4,16) | *Beta*(1,4) | *Beta*(4,16) | *Beta*(1,4) |
| 1 | 0.783 | -0.473 | 0.156 | 0.034 | 0.024 | 0.053 | 0.055 | 0.015 | -0.015 | 0.060 | 0.047 | 0.021 | 0.007 | 0.040 | 0.032 |
| 2 | 0.722 | 1.013 | 0.189 | 0.006 | -0.009 | 0.031 | 0.025 | -0.071 | -0.094 | -0.041 | -0.056 | -0.008 | -0.017 | 0.002 | -0.003 |
| 3 | 1.597 | -0.543 | 0.184 | 0.038 | 0.026 | 0.056 | 0.044 | -0.034 | -0.047 | -0.018 | -0.032 | -0.003 | -0.013 | 0.005 | -0.005 |
| 4 | 1.256 | 1.534 | 0.307 | -0.109 | -0.085 | -0.060 | -0.032 | -0.062 | -0.057 | -0.050 | -0.045 | -0.021 | -0.018 | -0.015 | -0.011 |
| 5 | 2.485 | 0.666 | 0.150 | -0.212 | -0.232 | -0.194 | -0.212 | -0.064 | -0.067 | -0.057 | -0.059 | -0.005 | -0.007 | -0.003 | -0.005 |
| 6 | 1.629 | -0.230 | 0.273 | -0.081 | -0.061 | -0.057 | -0.030 | -0.113 | -0.108 | -0.095 | -0.085 | -0.041 | -0.038 | -0.033 | -0.027 |
| 7 | 0.864 | -0.326 | 0.148 | 0.048 | 0.032 | 0.066 | 0.053 | 0.008 | -0.027 | 0.040 | 0.007 | 0.019 | 0.002 | 0.033 | 0.016 |
| 8 | 2.177 | -0.685 | 0.248 | -0.210 | -0.218 | -0.180 | -0.167 | -0.107 | -0.113 | -0.090 | -0.089 | -0.059 | -0.065 | -0.047 | -0.048 |
| 9 | 2.021 | -0.132 | 0.188 | 0.069 | 0.056 | 0.084 | 0.073 | -0.055 | -0.063 | -0.045 | -0.051 | -0.006 | -0.010 | -0.002 | -0.006 |
| 10 | 1.963 | 0.185 | 0.206 | -0.031 | -0.035 | -0.017 | -0.018 | -0.071 | -0.074 | -0.062 | -0.064 | -0.013 | -0.014 | -0.010 | -0.011 |
| 11 | 0.572 | 1.003 | 0.162 | 0.076 | 0.059 | 0.103 | 0.096 | -0.023 | -0.052 | 0.016 | 0.000 | 0.016 | 0.005 | 0.030 | 0.024 |
| 12 | 1.497 | 1.418 | 0.196 | -0.070 | -0.075 | -0.060 | -0.063 | -0.046 | -0.045 | -0.039 | -0.039 | -0.008 | -0.009 | -0.007 | -0.007 |
| 13 | 1.256 | 2.217 | 0.182 | -0.110 | -0.121 | -0.124 | -0.132 | 0.003 | 0.008 | 0.028 | 0.031 | -0.008 | -0.009 | -0.007 | -0.008 |
| 14 | 0.813 | -0.105 | 0.208 | 0.006 | 0.001 | 0.032 | 0.040 | -0.077 | -0.096 | -0.028 | -0.028 | -0.015 | -0.024 | 0.003 | 0.003 |
| 15 | 0.759 | 2.227 | 0.166 | 0.099 | 0.081 | 0.106 | 0.093 | -0.101 | -0.095 | -0.079 | -0.076 | 0.002 | -0.001 | 0.005 | 0.003 |
| 16 | 0.972 | 2.231 | 0.240 | -0.015 | -0.011 | 0.011 | 0.022 | -0.020 | -0.017 | -0.001 | -0.001 | -0.008 | -0.007 | -0.003 | -0.002 |
| 17 | 1.190 | -0.118 | 0.214 | -0.021 | -0.030 | -0.002 | -0.004 | -0.083 | -0.097 | -0.061 | -0.067 | -0.027 | -0.034 | -0.017 | -0.021 |
| 18 | 0.986 | 0.541 | 0.105 | 0.024 | -0.023 | 0.041 | 0.003 | -0.038 | -0.076 | -0.020 | -0.051 | 0.006 | -0.014 | 0.013 | -0.003 |
| 19 | 1.658 | -0.379 | 0.174 | 0.003 | -0.022 | 0.021 | 0.000 | -0.050 | -0.068 | -0.035 | -0.051 | -0.008 | -0.020 | -0.001 | -0.012 |
| 20 | 0.572 | -1.208 | 0.127 | 0.040 | 0.036 | 0.051 | 0.076 | 0.122 | 0.097 | 0.185 | 0.273 | 0.058 | 0.047 | 0.082 | 0.111 |

| Table C.12 Bias for item parameter estimate with 2000 examinees and 20 items | | | | | | | | | | | | | | | |
| --- | --- | --- | --- | --- | --- | --- | --- | --- | --- | --- | --- | --- | --- | --- | --- |
| Item | Generating | | | Bias for *a* | | | | Bias for *b* | | | | Bias for *c* | | | |
|  |  |  |  | Bayesian EMM | | BILOG-MG | | Bayesian EMM | | BILOG-MG | | Bayesian EMM | | BILOG-MG | |
|  | *a* | *b* | *c* | *Beta*(4,16) | *Beta*(1,4) | *Beta*(4,16) | *Beta*(1,4) | *Beta*(4,16) | *Beta*(1,4) | *Beta*(4,16) | *Beta*(1,4) | *Beta*(4,16) | *Beta*(1,4) | *Beta*(4,16) | *Beta*(1,4) |
| 1 | 1.050 | 1.351 | 0.321 | -0.079 | -0.055 | 0.002 | 0.030 | 0.008 | 0.016 | 0.037 | 0.046 | -0.020 | -0.016 | -0.007 | -0.002 |
| 2 | 2.437 | 0.413 | 0.182 | -0.098 | -0.107 | -0.089 | -0.097 | 0.017 | 0.016 | 0.026 | 0.025 | -0.005 | -0.006 | -0.004 | -0.004 |
| 3 | 2.213 | 1.507 | 0.153 | -0.235 | -0.249 | -0.232 | -0.245 | 0.047 | 0.048 | 0.054 | 0.055 | -0.003 | -0.004 | -0.003 | -0.003 |
| 4 | 1.157 | 1.291 | 0.192 | -0.049 | -0.057 | -0.036 | -0.041 | 0.023 | 0.022 | 0.034 | 0.033 | -0.008 | -0.009 | -0.005 | -0.007 |
| 5 | 1.488 | -0.960 | 0.172 | 0.003 | -0.017 | 0.009 | -0.020 | -0.004 | -0.024 | 0.004 | -0.028 | -0.014 | -0.030 | -0.007 | -0.033 |
| 6 | 1.152 | 0.796 | 0.166 | 0.022 | 0.011 | 0.034 | 0.025 | 0.024 | 0.019 | 0.036 | 0.032 | 0.001 | -0.002 | 0.004 | 0.001 |
| 7 | 0.581 | -0.678 | 0.176 | 0.013 | 0.008 | 0.030 | 0.042 | 0.027 | -0.001 | 0.101 | 0.119 | 0.005 | -0.005 | 0.031 | 0.036 |
| 8 | 1.917 | 0.341 | 0.178 | -0.054 | -0.063 | -0.044 | -0.053 | 0.017 | 0.015 | 0.027 | 0.025 | -0.003 | -0.005 | -0.001 | -0.002 |
| 9 | 0.861 | 0.894 | 0.223 | -0.014 | -0.016 | 0.010 | 0.014 | -0.016 | -0.021 | 0.009 | 0.010 | -0.013 | -0.014 | -0.004 | -0.004 |
| 10 | 1.160 | 0.389 | 0.239 | -0.031 | -0.027 | -0.014 | -0.005 | -0.018 | -0.016 | 0.001 | 0.007 | -0.013 | -0.011 | -0.007 | -0.004 |
| 11 | 1.094 | 0.242 | 0.278 | -0.048 | -0.034 | -0.019 | 0.003 | -0.025 | -0.014 | 0.008 | 0.026 | -0.023 | -0.018 | -0.011 | -0.003 |
| 12 | 0.332 | 0.089 | 0.175 | 0.031 | 0.027 | 0.071 | 0.114 | 0.083 | 0.043 | 0.329 | 0.506 | 0.016 | 0.007 | 0.071 | 0.111 |
| 13 | 0.557 | 0.308 | 0.151 | 0.041 | 0.028 | 0.064 | 0.060 | 0.071 | 0.037 | 0.132 | 0.114 | 0.024 | 0.011 | 0.043 | 0.036 |
| 14 | 2.248 | -0.622 | 0.279 | -0.319 | -0.307 | -0.271 | -0.230 | -0.096 | -0.095 | -0.076 | -0.065 | -0.063 | -0.062 | -0.049 | -0.041 |
| 15 | 1.931 | 0.853 | 0.242 | -0.094 | -0.087 | -0.085 | -0.077 | 0.019 | 0.021 | 0.028 | 0.030 | -0.006 | -0.005 | -0.005 | -0.004 |
| 16 | 0.551 | 0.661 | 0.138 | 0.038 | 0.019 | 0.060 | 0.046 | 0.068 | 0.025 | 0.115 | 0.079 | 0.018 | 0.002 | 0.033 | 0.020 |
| 17 | 0.860 | -0.283 | 0.137 | 0.014 | -0.006 | 0.023 | -0.002 | 0.028 | -0.012 | 0.050 | 0.000 | 0.005 | -0.015 | 0.014 | -0.010 |
| 18 | 1.072 | -1.032 | 0.110 | 0.046 | 0.032 | 0.050 | 0.026 | 0.076 | 0.049 | 0.087 | 0.038 | 0.041 | 0.022 | 0.049 | 0.016 |
| 19 | 0.481 | 1.099 | 0.214 | -0.003 | -0.012 | 0.053 | 0.065 | -0.042 | -0.072 | 0.066 | 0.068 | -0.021 | -0.030 | 0.013 | 0.015 |
| 20 | 1.100 | -1.004 | 0.126 | 0.047 | 0.033 | 0.053 | 0.033 | 0.069 | 0.044 | 0.082 | 0.042 | 0.031 | 0.014 | 0.039 | 0.012 |

# Appendix D

Table D.1. Item parameter recovery for flexMIRT data

| Item | Type | *a* | | | | | | *b* | | | | | | *c* | | | | | |
| --- | --- | --- | --- | --- | --- | --- | --- | --- | --- | --- | --- | --- | --- | --- | --- | --- | --- | --- | --- |
|  |  | BEMM | | BILOG | | flexMIRT | | BEMM | | BILOG | | flexMIRT | | BEMM | | BILOG | | flexMIRT | |
|  |  | *B*(4,16) | *B*(1,4) | *B*(4,16) | *B*(1,4) | *B*(4,16) | *B*(1,4) | *B*(4,16) | *B*(1,4) | *B*(4,16) | *B*(1,4) | *B*(4,16) | *B*(1,4) | *B*(4,16) | *B*(1,4) | *B*(4,16) | *B*(1,4) | *B*(4,16) | *B*(1,4) |
| 1 | value | 1.00 | 1.00 | 1.05 | 1.07 | 1.06 | 1.08 | 0.25 | 0.25 | 0.29 | 0.30 | 0.29 | 0.31 | 0.23 | 0.23 | 0.24 | 0.25 | 0.25 | 0.25 |
|  | SE | (0.06) | (0.06) | (0.16) | (0.17) | (0.17) | (0.20) | (0.03) | (0.03) | (0.10) | (0.10) | (0.10) | (0.11) | (0.01) | (0.01) | (0.04) | (0.04) | (0.04) | (0.05) |
| 2 | value | 0.77 | 0.77 | 0.76 | 0.75 | 0.76 | 0.75 | -2.07 | -2.09 | -2.09 | -2.16 | -2.10 | -2.17 | 0.17 | 0.16 | 0.17 | 0.12 | 0.17 | 0.12 |
|  | SE | (0.07) | (0.07) | (0.07) | (0.07) | (0.07) | (0.08) | (0.11) | (0.11) | (0.19) | (0.22) | (0.20) | (0.27) | (0.03) | (0.04) | (0.08) | (0.11) | (0.08) | (0.15) |
| 3 | value | 0.85 | 0.85 | 0.88 | 0.89 | 0.87 | 0.88 | 0.62 | 0.62 | 0.65 | 0.66 | 0.64 | 0.65 | 0.22 | 0.22 | 0.23 | 0.24 | 0.23 | 0.23 |
|  | SE | (0.07) | (0.06) | (0.14) | (0.15) | (0.15) | (0.17) | (0.04) | (0.04) | (0.10) | (0.10) | (0.10) | (0.11) | (0.01) | (0.01) | (0.04) | (0.04) | (0.04) | (0.04) |
| 4 | value | 0.74 | 0.74 | 0.77 | 0.78 | 0.76 | 0.77 | -0.44 | -0.45 | -0.37 | -0.33 | -0.39 | -0.35 | 0.20 | 0.19 | 0.22 | 0.24 | 0.22 | 0.23 |
|  | SE | (0.06) | (0.06) | (0.09) | (0.11) | (0.10) | (0.11) | (0.05) | (0.05) | (0.17) | (0.20) | (0.18) | (0.21) | (0.02) | (0.02) | (0.07) | (0.08) | (0.07) | (0.08) |
| 5 | value | 0.76 | 0.76 | 0.79 | 0.80 | 0.77 | 0.79 | 0.25 | 0.26 | 0.30 | 0.32 | 0.29 | 0.31 | 0.23 | 0.23 | 0.25 | 0.26 | 0.25 | 0.25 |
|  | SE | (0.06) | (0.06) | (0.11) | (0.12) | (0.12) | (0.12) | (0.04) | (0.04) | (0.13) | (0.14) | (0.14) | (0.14) | (0.02) | (0.02) | (0.05) | (0.05) | (0.05) | (0.05) |
| 6 | value | 0.84 | 0.84 | 0.84 | 0.82 | 0.84 | 0.82 | -1.30 | -1.31 | -1.30 | -1.37 | -1.30 | -1.37 | 0.16 | 0.15 | 0.17 | 0.13 | 0.17 | 0.13 |
|  | SE | (0.05) | (0.05) | (0.08) | (0.08) | (0.08) | (0.08) | (0.06) | (0.06) | (0.15) | (0.20) | (0.16) | (0.21) | (0.03) | (0.03) | (0.07) | (0.10) | (0.07) | (0.11) |
| 7 | value | 1.16 | 1.16 | 1.16 | 1.14 | 1.17 | 1.15 | -2.12 | -2.13 | -2.14 | -2.19 | -2.12 | -2.18 | 0.16 | 0.15 | 0.15 | 0.10 | 0.16 | 0.10 |
|  | SE | (0.07) | (0.07) | (0.10) | (0.10) | (0.12) | (0.12) | (0.09) | (0.09) | (0.14) | (0.15) | (0.15) | (0.18) | (0.04) | (0.04) | (0.07) | (0.09) | (0.08) | (0.13) |
| 8 | value | 0.63 | 0.63 | 0.63 | 0.63 | 0.62 | 0.62 | -1.15 | -1.17 | -1.13 | -1.14 | -1.15 | -1.19 | 0.18 | 0.17 | 0.19 | 0.19 | 0.19 | 0.17 |
|  | SE | (0.06) | (0.06) | (0.06) | (0.08) | (0.06) | (0.08) | (0.07) | (0.07) | (0.21) | (0.32) | (0.22) | (0.38) | (0.02) | (0.02) | (0.08) | (0.13) | (0.08) | (0.15) |
| 9 | value | 1.17 | 1.16 | 1.16 | 1.13 | 1.17 | 1.14 | -1.63 | -1.65 | -1.65 | -1.72 | -1.64 | -1.71 | 0.14 | 0.12 | 0.13 | 0.07 | 0.13 | 0.07 |
|  | SE | (0.06) | (0.06) | (0.10) | (0.09) | (0.11) | (0.11) | (0.06) | (0.06) | (0.11) | (0.11) | (0.12) | (0.13) | (0.03) | (0.03) | (0.06) | (0.06) | (0.07) | (0.09) |
| 10 | value | 0.82 | 0.82 | 0.81 | 0.80 | 0.81 | 0.79 | -1.58 | -1.60 | -1.60 | -1.69 | -1.60 | -1.69 | 0.16 | 0.14 | 0.15 | 0.10 | 0.15 | 0.10 |
|  | SE | (0.06) | (0.06) | (0.07) | (0.07) | (0.07) | (0.08) | (0.07) | (0.07) | (0.15) | (0.18) | (0.16) | (0.23) | (0.03) | (0.03) | (0.07) | (0.09) | (0.08) | (0.13) |
| 11 | value | 1.01 | 1.01 | 1.03 | 1.03 | 1.04 | 1.04 | 0.77 | 0.77 | 0.79 | 0.79 | 0.79 | 0.79 | 0.21 | 0.21 | 0.22 | 0.22 | 0.22 | 0.22 |
|  | SE | (0.07) | (0.07) | (0.17) | (0.17) | (0.19) | (0.20) | (0.04) | (0.04) | (0.08) | (0.08) | (0.08) | (0.08) | (0.01) | (0.01) | (0.03) | (0.03) | (0.03) | (0.03) |
| 12 | value | 0.89 | 0.88 | 0.91 | 0.92 | 0.91 | 0.91 | 0.31 | 0.30 | 0.34 | 0.34 | 0.33 | 0.33 | 0.19 | 0.19 | 0.20 | 0.20 | 0.20 | 0.20 |
|  | SE | (0.06) | (0.06) | (0.13) | (0.13) | (0.14) | (0.14) | (0.04) | (0.04) | (0.10) | (0.11) | (0.11) | (0.12) | (0.01) | (0.01) | (0.04) | (0.04) | (0.04) | (0.05) |

Table D.2. Item parameter recovery for IRTPRO data

| Type | Item | BEMM-C | | BEMM-P | | BILOG | | IRTPRO | |
| --- | --- | --- | --- | --- | --- | --- | --- | --- | --- |
|  |  | *B*(4,16) | *B*(1,4) | *B*(4,16) | *B*(1,4) | *B*(4,16) | *B*(1,4) | *B*(4,16) | *B*(1,4) |
| *a* | 1 | 0.58 | 0.58 | 0.58 | 0.58 | 0.56 | 0.57 | 0.46 | 0.46 |
|  |  | (0.14) | (0.14) | (0.05) | (0.06) | (0.13) | (0.14) | (0.27) | (0.27) |
|  | 2 | 0.51 | 0.51 | 0.52 | 0.52 | 0.56 | 0.62 | 0.48 | 0.48 |
|  |  | (0.11) | (0.11) | (0.09) | (0.09) | (0.13) | (0.19) | (0.24) | (0.25) |
|  | 3 | 0.71 | 0.71 | 0.71 | 0.70 | 0.73 | 0.77 | 0.65 | 0.61 |
|  |  | (0.10) | (0.09) | (0.10) | (0.10) | (0.22) | (0.28) | (0.37) | (0.36) |
|  | 4 | 0.47 | 0.47 | 0.48 | 0.47 | 0.52 | 0.55 | 0.44 | 0.43 |
|  |  | (0.12) | (0.12) | (0.08) | (0.08) | (0.11) | (0.15) | (0.22) | (0.23) |
|  | 5 | 0.48 | 0.48 | 0.48 | 0.48 | 0.51 | 0.52 | 0.40 | 0.40 |
|  |  | (0.13) | (0.13) | (0.06) | (0.05) | (0.11) | (0.12) | (0.23) | (0.23) |
| *b* | 1 | -2.64 | -2.64 | -2.60 | -2.59 | -2.70 | -2.67 | -3.18 | -3.16 |
|  |  | (0.31) | (0.31) | (0.13) | (0.13) | (0.54) | (0.63) | (0.94) | (0.99) |
|  | 2 | -0.75 | -0.75 | -0.73 | -0.73 | -0.65 | -0.37 | -0.80 | -0.84 |
|  |  | (0.12) | (0.12) | (0.10) | (0.10) | (0.31) | (0.58) | (0.35) | (0.57) |
|  | 3 | 0.21 | 0.21 | 0.22 | 0.21 | 0.30 | 0.37 | 0.19 | 0.08 |
|  |  | (0.07) | 0.00 | (0.07) | (0.07) | (0.24) | (0.34) | (0.23) | (0.34) |
|  | 4 | -1.23 | -1.23 | -1.20 | -1.20 | -1.10 | -0.88 | -1.31 | -1.34 |
|  |  | (0.16) | (0.16) | (0.11) | (0.11) | (0.34) | (0.63) | (0.44) | (0.65) |
|  | 5 | -2.26 | -2.26 | -2.22 | -2.22 | -2.15 | -2.07 | -2.62 | -2.64 |
|  |  | (0.28) | (0.28) | (0.13) | (0.11) | (0.46) | (0.62) | (0.81) | (0.94) |
| *c* | 1 | 0.19 | 0.19 | 0.21 | 0.22 | 0.20 | 0.22 | 0.20 | 0.21 |
|  |  | (0.04) | (0.04) | (0.06) | (0.07) | (0.09) | (0.19) | (0.09) | (0.18) |
|  | 2 | 0.19 | 0.19 | 0.20 | 0.20 | 0.22 | 0.31 | 0.19 | 0.18 |
|  |  | (0.02) | (0.02) | (0.03) | (0.04) | (0.09) | (0.18) | (0.09) | (0.17) |
|  | 3 | 0.19 | 0.18 | 0.19 | 0.19 | 0.22 | 0.24 | 0.17 | 0.14 |
|  |  | (0.03) | (0.01) | (0.02) | (0.02) | (0.08) | (0.12) | (0.08) | (0.12) |
|  | 4 | 0.19 | 0.19 | 0.21 | 0.21 | 0.22 | 0.29 | 0.20 | 0.19 |
|  |  | (0.02) | (0.02) | (0.04) | (0.04) | (0.09) | (0.20) | (0.09) | (0.17) |
|  | 5 | 0.19 | 0.19 | 0.21 | 0.21 | 0.21 | 0.24 | 0.20 | 0.20 |
|  |  | (0.04) | (0.04) | (0.05) | (0.03) | (0.09) | (0.20) | (0.09) | (0.18) |

Figure D.1.

Item parameter recovery for flexMIRT data

Figure D.2.

Item parameter recovery for IRTPRO data
